# Metabolic and Lipidomic Markers Differentiate COVID-19 From Non-Hospitalized and Other Intensive Care Patients

**DOI:** 10.3389/fmolb.2021.737039

**Published:** 2021-12-02

**Authors:** Franziska Schmelter, Bandik Föh, Alvaro Mallagaray, Johann Rahmöller, Marc Ehlers, Selina Lehrian, Vera von Kopylow, Inga Künsting, Anne Sophie Lixenfeld, Emily Martin, Mohab Ragab, Roza Meyer-Saraei, Fabian Kreutzmann, Ingo Eitel, Stefan Taube, Nadja Käding, Eckard Jantzen, Tobias Graf, Christian Sina, Ulrich L. Günther

**Affiliations:** ^1^ Institute of Nutritional Medicine, University of Lübeck, Lübeck, Germany; ^2^ Research and Development Department, GALAB Laboratories GmbH, Hamburg, Germany; ^3^ Medical Department I, University Hospital Schleswig-Holstein, Lübeck, Germany; ^4^ Institute of Chemistry and Metabolomics, University of Lübeck, Lübeck, Germany; ^5^ Department of Anesthesiology and Intensive Care, University Medical Center Schleswig-Holstein, Lübeck, Germany; ^6^ Department of Cardiology, Angiology and Intensive Care Medicine, University Heart Center Lübeck, Lübeck, Germany; ^7^ German Centre for Cardiovascular Research (DZHK), Partner Site Hamburg/Kiel/Lübeck, Lübeck, Germany; ^8^ Institute of Virology and Cell Biology, University of Lübeck, Lübeck, Germany; ^9^ Department of Infectious Diseases and Microbiology, University of Lübeck, Lübeck, Germany

**Keywords:** NMR, metabolomics, SARS-CoV-2, COVID-19, lipoproteins

## Abstract

Coronavirus disease 2019 (COVID-19) is a viral infection affecting multiple organ systems of great significance for metabolic processes. Thus, there is increasing interest in metabolic and lipoprotein signatures of the disease, and early analyses have demonstrated a metabolic pattern typical for atherosclerotic and hepatic damage in COVID-19 patients. However, it remains unclear whether this is specific for COVID-19 and whether the observed signature is caused by the disease or rather represents an underlying risk factor. To answer this question, we have analyzed 482 serum samples using nuclear magnetic resonance metabolomics, including longitudinally collected samples from 12 COVID-19 and 20 cardiogenic shock intensive care patients, samples from 18 severe acute respiratory syndrome coronavirus 2 (SARS-CoV-2) antibody-positive individuals, and single time point samples from 58 healthy controls. COVID-19 patients showed a distinct metabolic serum profile, including changes typical for severe dyslipidemia and a deeply altered metabolic status compared with healthy controls. Specifically, very-low-density lipoprotein and intermediate-density lipoprotein particles and associated apolipoprotein B and intermediate-density lipoprotein cholesterol were significantly increased, whereas cholesterol and apolipoprotein A2 were decreased. Moreover, a similarly perturbed profile was apparent when compared with other patients with cardiogenic shock who are in the intensive care unit when looking at a 1-week time course, highlighting close links between COVID-19 and lipid metabolism. The metabolic profile of COVID-19 patients distinguishes those from healthy controls and also from patients with cardiogenic shock. In contrast, anti-SARS-CoV-2 antibody-positive individuals without acute COVID-19 did not show a significantly perturbed metabolic profile compared with age- and sex-matched healthy controls, but SARS-CoV-2 antibody-titers correlated significantly with metabolic parameters, including levels of glycine, ApoA2, and small-sized low- and high-density lipoprotein subfractions. Our data suggest that COVID-19 is associated with dyslipidemia, which is not observed in anti-SARS-CoV-2 antibody-positive individuals who have not developed severe courses of the disease. This suggests that lipoprotein profiles may represent a confounding risk factor for COVID-19 with potential for patient stratification.

## Introduction

Although severe acute respiratory syndrome coronavirus 2 (SARS-CoV-2) is a primarily respiratory virus, it has become evident that it does not exclusively affect the airways and lungs but a multitude of organs throughout the body (Prasad and Prasad, 2020; Zaim et al., 2020). Accordingly, severe cases of coronavirus disease 2019 (COVID-19) are often characterized by multiorgan damage. In the lungs, COVID-19 causes acute respiratory distress arising from inflammatory cell infiltration dominated by lymphocytes, diffuse alveolar damage, and pulmonary edema (Xu et al., 2020a). Moreover, COVID-19 patients show neurological symptoms (Mao et al., 2020) and renal (Benedetti et al., 2020; Li et al., 2020; Ronco et al., 2020), and liver damage (Xu et al., 2020b). Vascular damage and thromboembolisms contribute to the observed multiorgan damage and overall lethality of the disease (Nishiga et al., 2020).

This raises the question of whether there is a common underlying molecular mechanism that is reflected in metabolic profiles. Early proteomics studies showed dysregulation of coagulative and proinflammatory pathways associated with metabolic suppression and dyslipidemia linked to the clinical severity of the disease (Shen et al., 2020; Wu et al., 2020). Metabolic profiles from COVID-19 patients were shown to be distinctly different from those of controls, with metabolic markers indicative of liver dysfunction, dyslipidemia, diabetes, and coronary heart disease risk (Kimhofer et al., 2020). In a Spanish cohort of hospitalized COVID-19 patients, increased ketone bodies and markers of dyslipidemia were reported (Bruzzone et al., 2020). Lipoprotein analyses showed increased very-low-density lipoprotein (VLDL) and intermediate-density lipoprotein (IDL) (sub)-fractions and decreased high-density (HDL) levels in both studies (Bruzzone et al., 2020; Kimhofer et al., 2020). In a preliminary report, we have previously reported a very similar signature derived from nuclear magnetic resonance (NMR) metabolomics (Schmelter et al., 2021) characterized by increased levels of glutamic acid, phenylalanine, and glucose, which is consistent with the findings of the two other studies (Bruzzone et al., 2020; Kimhofer et al., 2020). These results seem to underline systemic defects associated with COVID-19 and demonstrate the suitability of metabolomics for the detection and potentially stratification of COVID-19 patients.

However, it is crucially important to identify whether the associated differences in metabolic profiles are specific for COVID-19 and whether markers can be identified that distinguish COVID-19 from other diseases that affect cardiovascular parameters. It is equally important to identify whether metabolic and lipoprotein markers are consistent with those reported for other previously reported patient cohorts (Bruzzone et al., 2020; Kimhofer et al., 2020). Moreover, we have analyzed samples from individuals who tested positive for COVID-19 without developing a severe form of the disease to determine whether our observed signature is specific for patients who have developed COVID-19 or also for individuals who were infected by the virus without developing severe disease.

Here, we have utilized NMR metabolomics to analyze the metabolic and lipoprotein profiles of COVID-19 patients hospitalized at the intensive care unit (ICU) of the University Hospital of Schleswig-Holstein (Campus Lübeck) compared with a group of healthy controls (HCs) and a cohort of cardiogenic shock (CS) patients treated in the same ICU but tested negative for SARS-CoV-2. We used an analysis of variance–simultaneous component analysis (ASCA) time-course analysis (Smilde et al., 2005) to analyze longitudinally sampled blood taken daily for 1 week to identify patient-specific changes over time. Our results show a serum metabolomics profile with severe dyslipidemia in COVID-19 patients compared with HC. This profile also distinguishes COVID-19 from CS consistently over a time course of 7 days.

In addition, we have analyzed samples from a different cohort of asymptomatic individuals who tested positive for anti-SARS-CoV-2 antibodies (anti-S1-IgG+) compared with antibody-negative individuals. Antibody titers of anti-SARS-CoV-2 antibody-positive individuals correlated with markers of metabolic health.

## Materials and Methods

### Study Participants

All participants provided written informed consent according to the declaration of Helsinki. ICU patients with either COVID-19 or CS were included within the LUERIC study at the University Hospital of Schleswig-Holstein (Campus Lübeck) after evaluation and approval by the corresponding ethics committee (LUERIC-MICROBIOME Nr. 19-019 and 19-019 A, University of Lübeck). COVID-19 status was determined by a reverse transcription-polymerase chain reaction from nasopharyngeal swabs and typical symptoms (fever, cough, and dyspnea). In the ICU, serum samples were collected twice daily, once between 7 and 9 am and once between 7 and 9 pm.

Non-hospitalized individuals were recruited by a population-based SARS-CoV-2 monitoring cohort approved by the corresponding ethics committee [enzyme-linked immunoassay (ELISA) Nr. 20-150, University of Lübeck]. This cohort included 18 individuals who tested positive for Anti-SARS-CoV-2 antibodies along with 58 individuals who tested negative using commercially available ELISA kits. Acute infection was determined by a reverse transcription-polymerase chain reaction from nasopharyngeal swabs on the same day of serum sampling before subsequent antibody analysis.

### NMR-Metabolomics

Aliquots of the frozen serum samples were thawed at room temperature for several minutes. Samples were mixed 1/1 (v/v) with 75 mM sodium phosphate buffer (pH 7.4) and shaken manually for 1 min, and 600 µl of mixed samples were transferred into a 5-mm NMR tube. NMR analysis was performed on Bruker 600 MHz Avance III HD spectrometer with a TXI probe. A Bruker SampleJet automatic sample changer was used, and cooling was set at 6°C. All experiments were recorded using the Bruker *in-vitro* Diagnostic Research (IVDr) protocol. For quality control, the NMR spectrometer was calibrated daily using a strict standard operating procedure as described by Dona et al. (2014).

Four different ^1^H NMR experiments were measured per sample at a temperature of 310K. One-dimensional (1D) proton spectra were recorded using a standard Bruker pulse program (noesygppr1d) with presaturation water suppression during the 4-s recycle delay. A 1D Carr–Purcell–Meiboom–Gill spin-echo experiment (pulse program: cpmgpr1d) was acquired for the suppression of proteins and other macromolecular signals. These spectra were acquired with 32 scans and 131,072 data points. Furthermore, 2D J-resolved and diffusion-edited experiments were performed. From these spectra, concentrations of 39 metabolites and 112 lipoproteins were obtained automatically using Bruker Quantification in plasma/serum B.I.Quant-PS 2.0.0 and Bruker IVDr Lipoprotein Subclass Analysis B.I.-LISA (Bruker BioSpin).

### Anti-SARS-CoV-2 ELISA

Antibody-titers against SARS-CoV-2 in serum samples from non-hospitalized individuals were determined using ELISAs for the spike (S1) and nucleocapsid (NCP) antigens following the manufacturer’s protocols (anti-SARS-CoV-2-ELISA IgG, EI 2606-9601G; anti-SARS-CoV-2-NCP-ELISA IgG, EI 2606-9601-2G; EUROIMMUN Medizinische Labordiagnostika AG, Germany). Individuals were categorized as positive (anti-S1-IgG+) if they reached a reference value of 0.6 compared with a standard serum. The determination of IgG antibody-titers was repeated after a 6-week interval for patients who initially tested positive, combined with additional NMR metabolomics.

### Statistics

Primary NMR data were processed, scaled, and aligned using Matlab (TheMathworks) and MetaboLab (Günther et al., 2000; Ludwig and Günther, 2011). Principal component analysis (PCA) and partial least squares-discriminant analysis (PLS-DA) were calculated using PLS-Toolbox (Eigenvector Research) in Matlab. PCA was applied to variance-scaled and mean-centered concentration values of metabolites and lipoproteins determined by Bruker’s IVDr software. Similarly, PLS-DA was calculated using PLS-Toolbox using Venetian blinds for cross-validation and subsequent calculation of the area under the receiver operating characteristic (ROC) curve. To analyze longitudinal data, we used ASCA in PLS-Toolbox (Smilde et al., 2005), which allows us to simultaneously consider different factors such as changes over time and changes between cohorts of patients. For pairwise comparisons, means of the longitudinal measurements were calculated for each ICU patient and compared with the respective controls. Pairwise comparisons were carried out using multiple, unpaired t-tests adjusted for multiple comparisons using the false discovery rate approach in Prism 9.1.0 (GraphPad Software, LLC). Spearman correlation plots and Forest plots were also generated using GraphPad Prism. Metabolic parameters were evaluated in a subgroup of anti-S1-IgG+ individuals using correlation plots of parameters against SARS-CoV-2 titers in Matlab. Specifically, pairwise correlations were generated using the *corr* function provided by the Matlab Statistics Toolbox using Spearman type ranked correlations.

## Results

### Study Population

**FIGURE 1:**
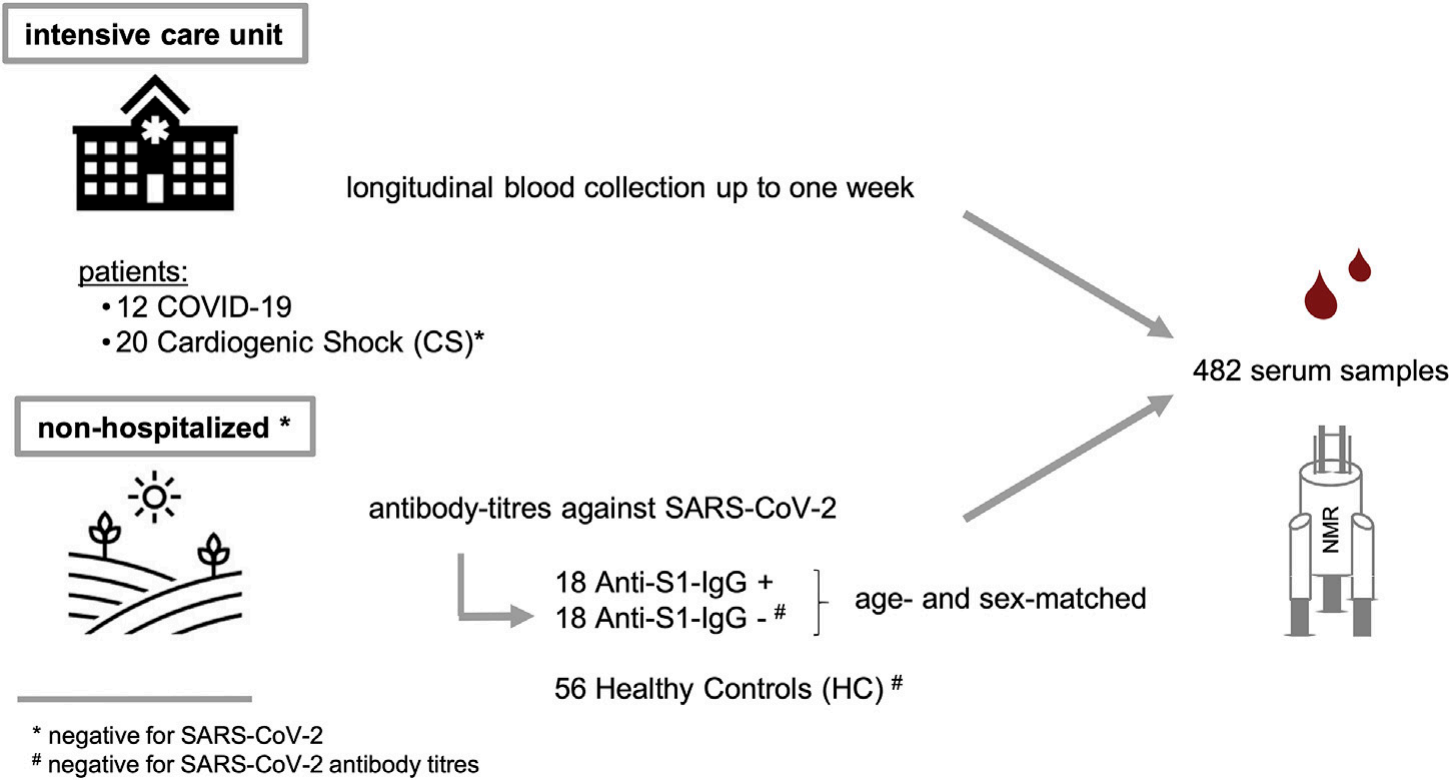
Study design and description of two sample cohorts. (i) A critically ill cohort from intensive care unit (COVID-19 and CS) and a non-hospitalized group (SARS-CoV-2 positive and negative).

Two different study populations were examined as summarized in Figure 1 and Table 1. The first group consisted of patients from the same ICU with either COVID-19 or CS; the latter were SARS-CoV-2 RNA-negative. ICU samples were collected longitudinally for 7 days, with one sample taken in the morning, the other in the evening. The mean age of COVID-19 patients was 63.5 ± 9.1 years and did not differ significantly from CS patients (CS: 69.1 ± 16.0 years, *p* = 0.277). The study groups had similar average body mass indexes (BMIs) of 28.7 ± 6.4 kg/m^2^ for COVID-19 and 25.7 ± 3.1 kg/m^2^ for CS (*p* = 0.088). The Simplified Acute Physiology Score II (SAPS II) was calculated for COVID-19 and CS patients in the ICU to classify disease severity, and no significant differences were found (*p* = 0.182). However, 17% of COVID-19 patients and one-quarter of CS patients had a fatal outcome during or a few weeks after the ICU.

**TABLE 1:** Study population metrics.

			Total	Sex			Age (years)	BMI (kg/m^2^)	SAPS II score
				Female	Male				
Intensive care unit	COVID-19	*n*	12	6	6	mean	63.5	28.7	42.1
		%		50	50	SD	9.1	6.4	12.0
	Cardiogenic Shock (CS)	*n*	20	4	16	mean	69.1	25.7	48.7
		%		20	80	SD	16.0	3.1	13.8
		COVID-19 vs. CS				*p*-value	0.277	0.088	0.182

			Total	Sex			Age (years)	BMI (kg//m^2^)	Anti-S1 IgG titer
				Female	Male				
Non-hospitalized	Healthy Control (HC)	*n*	58^a^	26	30	mean	51.0	25.2	—
		%		46.4	53.6	SD	15.8	4.6	—
	Anti-S1-IgG+	*N*	18	8	10	mean	44.1	26.7	2.44
		%		44.4	55.6	SD	15.9	5.2	1.70
	Anti-S1-IgG-	*N*	18	8	10	mean	44.1	25.2	—
		%		44.4	55.6	SD	15.9	4.1	—
		COVID-19 vs. HC				*p*-value	0.010	0.030	—
		Anti-S1-IgG+ vs. Anti-S1-IgG-				*p*-value	>0.999	0.342	—

a Two subjects unknown.

A second population consisting of non-hospitalized individuals who tested negative for SARS-CoV-2 was used as HCs. The mean age of COVID-19 patients was significantly higher than in the HC group (HC: 51.0 ± 15.8 years, *p* = 0.010), and they had a somewhat lower average BMI value of 25.2 ± 4.6 kg/m^2^ (*p* = 0.030).

Additionally, we included a group of non-hospitalized individuals who tested positive for anti-SARS-CoV-2 antibodies (anti-S1-IgG+) and compared them to age- and sex-matched subgroups of HCs (anti-S1-IgG-) who were tested negative for SARS-CoV-2 RNA on the same day. Anti-S1-IgG+ and Anti-S1-IgG- were also matched regarding their BMI values (anti-S1-IgG+: 26.7 ± 5.2 kg/m^2^, anti-S1-IgG-: 25.2 ± 4.1 kg/m^2^, *p* = 0.342).

### NMR Metabolomics and Lipidomics Profile of COVID-19, HC, and ICU CS Patients

**FIGURE 2:**
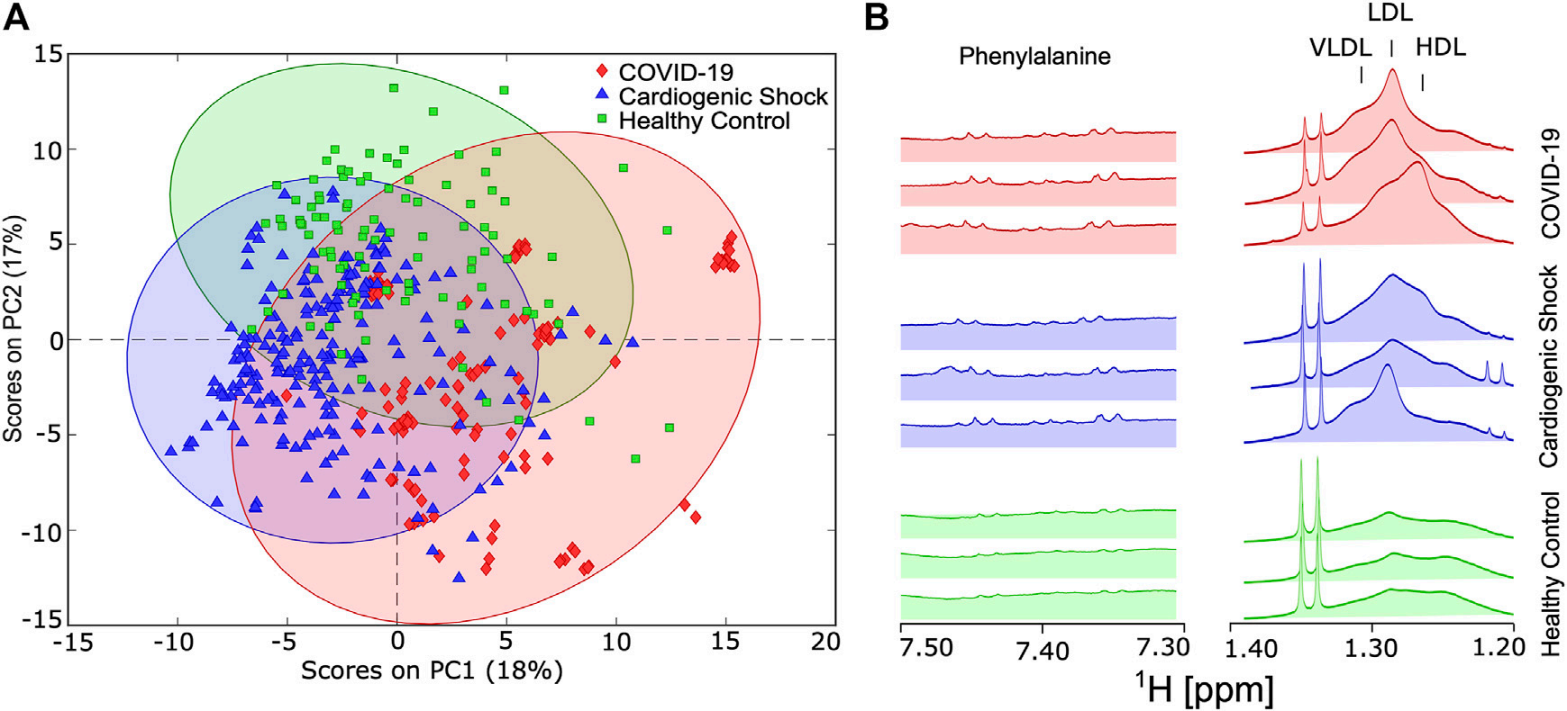
**(A)** Score plot of principal component analysis using variance-scaled and mean-centered IVDr NMR data for longitudinally collected serum samples from COVID-19 patients (red) and cardiogenic shock (CS) patients (blue), as well as one-time samples from healthy controls (HC) (green). **(B)** Key sections of 1D-NOESY spectra showing typical differences between HC, CS, and COVID-19 group.

To test whether there are distinct metabolic changes in SARS-CoV-2-infected patients, NMR spectra from blood samples of COVID-19, CS, and HC were acquired. PCA was carried out for approximately 150 parameters (39 metabolites, 112 lipoprotein-related parameters, listed in Supplementary Table S1) derived from the IVDr analysis for all three groups. Principal component (PC) 1 accounted for 18% of the total variation and discriminated between longitudinally acquired COVID-19 samples and both HCs and CS patients, whereas PC2 accounted for 17% of the total variation. PCA plots of variance-scaled and mean-centered Bruker IVDr data showed reasonably good separation between groups of ICU patients with COVID-19 (diamond in red) and HCs (square in green). The separation between ICU patients either with COVID-19 or CS (triangle in blue) is also a group, but the separation of the PCA clusters is not perfect (Figure 2A).

Visible differences between the groups were also readily observed in key regions 1D-NOESY spectra, as shown in Figure 2B.

### Unsupervised Analysis Reveals Metabolite and Lipoprotein Clusters Distinguishing COVID-19 From HC and ICU CS Patients

**FIGURE 3:**
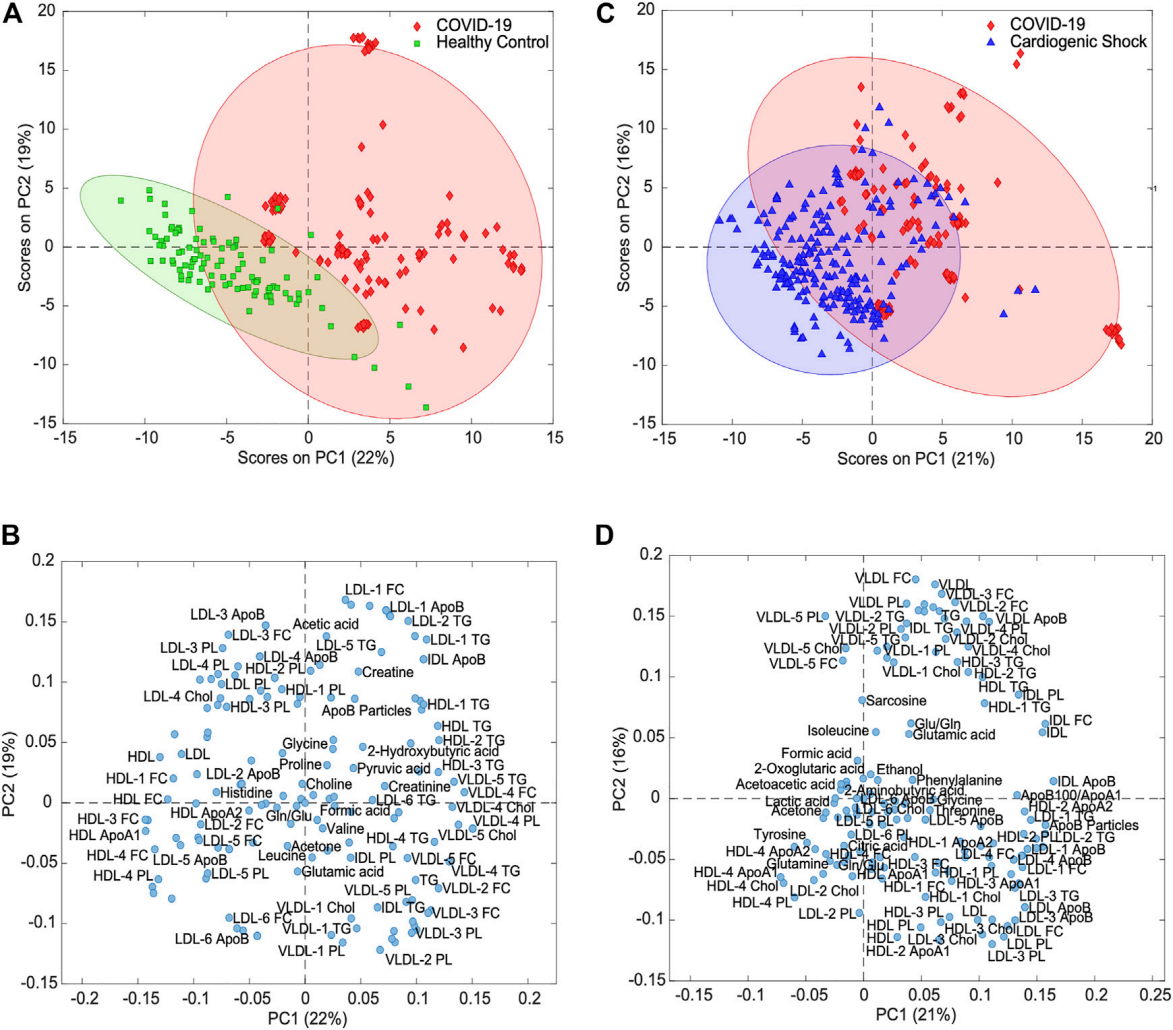
Principal component analysis for variance-scaled and mean-centered IVDr NMR data. **(A)** Scores plot of COVID-19 samples (red) *vs.* healthy controls (green) showing good separation of two groups. **(B)** Loadings plot of same PCA showing selection of metabolites and lipoproteins. **(C)** PCA scores plot of COVID-19 (red) vs. cardiogenic shock samples (blue) showing partial separation of two groups. **(D)** Loadings plot of same PCA showing groups of correlated metabolites and lipoproteins.

To identify metabolites and lipoproteins that are different in COVID-19 patients, we carried out pairwise PCAs of both COVID-19 and HCs and COVID-19 and CS. Figure 3A shows variance-scaled and mean-centered PCA, confirming a good separation of COVID-19 patients (diamond in red) from HCs (square in green), mainly by PC1 with a total variation of 22%. The loading plot for PC1 revealed several clusters of lipoprotein fractions and subfractions that were most influential for group separation (Figure 3B).

To evaluate the specificity of metabolic changes in COVID-19 patients, the metabolic profiles were compared with those from a group of SARS-CoV-2-negative ICU patients with respiratory distress due to CS. Unsupervised PCA of the targeted analysis confirmed the partial separation in the first two PCs (Figure 3C). The loadings plot again showed lipoprotein clusters that separated COVID-19 from CS (Figure 3D), in large parts recapitulating the relevant class indicators for separating COVID-19 and HCs (Figure 3B).

The level of separation was further tested by PLS-DA with cross-validation and subsequent calculation of ROC curves, which gave areas under the ROC curve of 99 and 98% for COVID-19 vs. HC and for COVID-19 *vs.* CS, respectively (Supplementary Figure S1). These levels are like too high, considering that the data used here consisted of longitudinally collected samples of the same patients.

### Supervised Analysis Distinguishes COVID-19 from HC and ICU CS Patients

**FIGURE 4:**
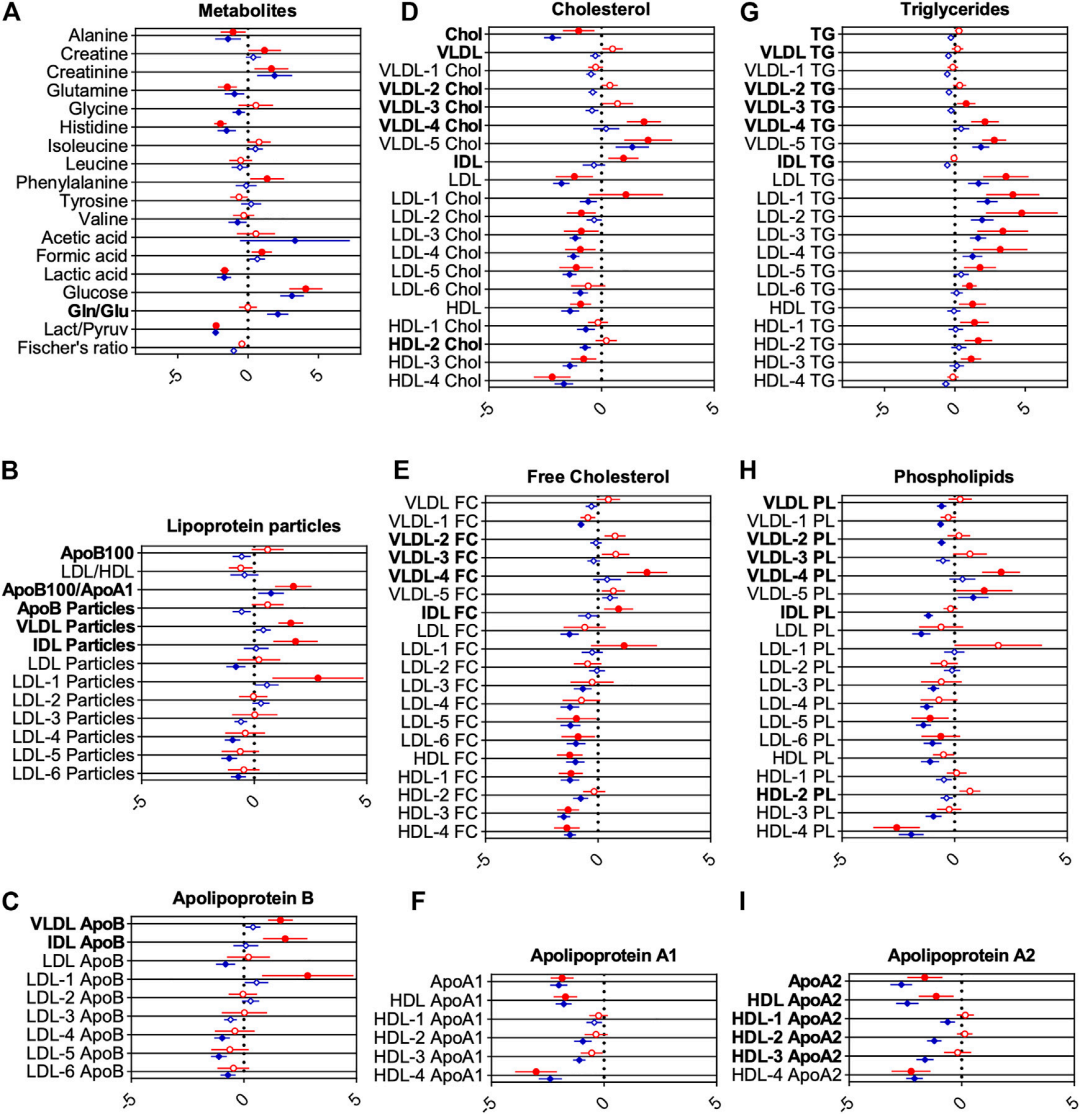
Forest plots showing changes for groups of metabolites **(A)** and lipoprotein classes **(B-I)**. Middle line indicates reference average, whereas circles and diamonds on horizontal axes show changes scaled by standard deviations. Red circles show changes for COVID-19 samples, blue circles for CS, both with healthy controls as reference (vertical line). Statistically significant differences between CS and COVID-19 to HC determined using false discovery method of Benjamini, Krieger, and Yekutieli (Q = 1%) are indicated by filled circles or diamonds. Letters printed in bold indicate metabolites and lipoproteins, which are significantly changed between COVID-19 and CS.

To examine the metabolic effects of COVID-19 more closely, pairwise analyses of metabolites and lipoproteins were carried out. For statistical significance, multiple t-tests using the false discovery method of Benjamini, Krieger, and Yekutieli (Q = 1%) were calculated (Supplementary Table S1). Forest plots (Figure 4; Supplementary Figures S2, S3) show relative deviation from the average of HCs as reference. The filled red circles indicate significant changes between COVID-19 and HCs, whereas the filled blue diamonds show significant changes between CS and HCs.

The reference line at 0 indicates no relative deviation compared with HC. This enables an easy way to compare metabolic differences to HCs for both ICU groups. Metabolites and lipoproteins, which were significantly changed between COVID-19 and CS, are labeled with bold letters.

### Metabolic Markers for COVID-19 Compared With HC

For COVID-19, glucose and formic acid levels were increased, and lactic acid and the lactic acid/pyruvic acid ratio decreased compared with HCs, indicating a disturbed energy status. Decreased alanine, glutamine, and histidine may be indicative of disrupted hepatic amino acid metabolism and hepatic damage (Kimhofer et al., 2020). Increased creatine, creatinine, and phenylalanine levels compared with HCs further support a disrupted hepatic or renal metabolism.

Lipoprotein profiles of COVID-19 patients showed markers of severe dyslipidemia when compared with HCs. The ratio of apolipoprotein B100 to apolipoprotein A1 (ApoB100/ApoA1), a strong independent risk factor for cardiovascular diseases, was significantly increased (Figure 4B). VLDL, IDL, and large-sized low-density lipoprotein (LDL)-1 particles were increased as well (Figure 4B). Triglycerides for nearly all lipoprotein subfractions were distinctly elevated for COVID-19, most pronounced for LDL (Figure 4G). HDL, LDL, several subfractions of HDL and LDL cholesterol (Figures 4D,E), and phospholipids (Figure 4H) were decreased (especially for smaller size particles including LDL-3, 4, 5, and 6, with the largest effects observed for HDL-4 cholesterol and HDL-4 phospholipids). Subfraction analysis of VLDL lipoproteins further revealed that triglycerides, phospholipids, esterified, and free cholesterol bound to lipoproteins of smaller sizes (VLDL-3, 4, and 5) were increased dramatically (Figures 4D,E,G,H). Accordingly, levels of apolipoproteins A1 and ApoA2 in HDL and smaller-sized HDL were reduced (largest effects for HDL-4 ApoA1 and A2) (Figures 4F,I), whereas VLDL, IDL, and LDL-1 ApoB were increased (Figure 4C).

### Metabolic Markers for CS Compared With HC

For critically ill CS patients, levels of several amino acids (alanine, glutamine, glycine, histidine, and valine) and the level of lactic acid and the ratio of lactic/pyruvic acid were decreased in comparison with HCs. Creatinine, acetic acid, glucose, and the glutamine/glutamic acid ratio were increased (Figure 4A). The ApoB100/apoA1 ratio remains increased, whereas LDL particle numbers were slightly decreased for CS patients (Figure 4B). Similar to the profile in COVID-19 patients, an altered triglyceride profile was observed, but in contrast to the COVID-19 patients, only LDL levels were increased for CS (Figure 4G). LDL and HDL in cholesterol (Figures 4D,E) and phospholipids (Figure 4H) remain decreased for CS patients. Several subfractions of VLDL phospholipids appear to be decreased for CS patients (Figure 4H). We also found decreased IDL phospholipids, although this parameter is prone to artifacts owing to the low concentration of IDL particles (Figure 4H). For apolipoprotein A1 and A2, a significant decrease in nearly all fractions was found for CS patients (Figure 4F,I). LDL ApoB and smaller-sized particles of LDL ApoB were decreased (Figure 4C).

### Differences in Lipoproteins Between COVID-19 and CS

Importantly, COVID-19 displayed dyslipidemic changes in comparison with ICU CS patients with similar markers, as shown in Figure 4. However, we observed several deviations from HCs that were different for the two groups. In particular, ApoB, VLDL, and IDL particles (Figure 4B), smaller-sized VLDL fractions (VLDL-2, -3, and -4), and IDL-bound lipids in cholesterol, triglycerides, and phospholipids were significantly different for COVID-19 compared with CS (Figures 4D,E,G,H). Moreover, ApoB bound to VLDL and IDL were further increased in COVID-19 patients (Figure 4C). Apolipoprotein A2 and ApoA2 bound to large-sized HDL were significantly different in COVID-19 patients compared with CS (Figure 4I).

Interestingly, for cholesterol, total apolipoprotein A2, and apolipoprotein A2 bound to HDL, stronger effects were observed for CS patients in comparison with COVID-19 and HCs (Figures 4D,I). Together, these results indicate a disturbed energy status and possibly hepatic damage in COVID-19 patients.

### ASCA Together With Selected Lipoprotein Time Courses Show a Strong Signature Over Time

**FIGURE 5:**
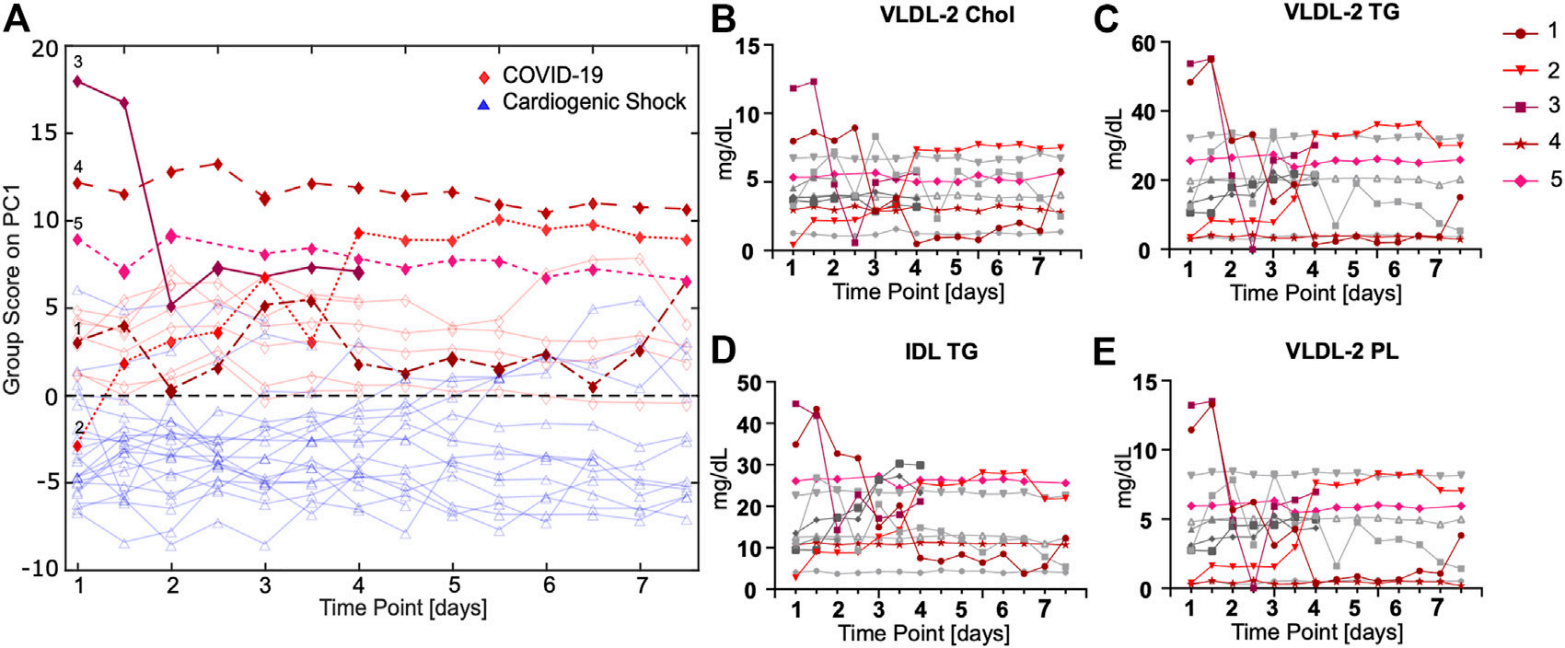
**(A)** ASCA analysis of COVID-19 (red) *vs.* CS (blue) samples shows a separation between groups in consideration of different groups and time points. **(B–E)** Time courses of lipoproteins measured twice a day for COVID-19 patients that are only significant for comparison of COVID-19 *vs.* CS show striking progressions over time for some patients (highlighted in red).

To evaluate time courses for longitudinal samples taken twice a day, an ASCA for all ICU patients was performed. Five COVID-19 cases were highlighted as examples, which will be discussed in further detail. ASCA group samples are shown with respect to time and the largest variations (Figure 5A). ASCA was supported by individually monitoring selective markers (Supplementary Figure S4). For some patients, we observed big changes over time; for others, the ASCA score remains largely unchanged. Interestingly, COVID-19 and CS patients can be clearly distinguished even when taking the time course into account. The underlying signature largely points toward lipoprotein profiles.

The ASCA for COVID-19 patients reveals an unexpected time course for lipoproteins for some but not all subjects (Figures 5B–D; Supplementary Figure S5). For patient 1, a slightly overweight (BMI 28 kg/m^2^) 56-year-old male with allergic asthma, significant fluctuations in the lipoproteins were observed. From time point six onward, a marked decrease to a constantly low lipoprotein level was observed. This correlates with the onset of dialysis treatment. The patient had a fatal outcome several days after the last blood sample was taken. For patient 2, extremely low lipoprotein concentrations were determined at the beginning of treatment, which significantly increased during the time course, especially from time point seven. This patient was transferred from France, where he had been treated with chloroquine, for which an LDL lowering effect has previously been reported (Sachet et al., 2007). We attribute the increase in LDL and VLDL fractions to the nutritional regime applied for this patient who had a particularly low BMI of 24.5 kg/m^2^. Patients 3, a relatively young woman (47 years old) in relation to the study cohort, had very high BMI values (>30 kg/m^2^), which may have influenced their lipoprotein profile, but apart from obesity, no other comorbidities. The normal-weight patient 4 (BMI 22 kg/m^2^) with Parkinson's disease had an unremarkable clinical course regarding COVID-19. The lipoprotein level of this patient remained constant at a low value. Lipoprotein levels remain constant for patient 5, who had an extremely high BMI (>35 kg/m^2^) and, in addition to severe obesity, arterial hypertension.

### Anti-SARS-CoV-2 Antibody-Titers Correlate With Metabolomic and Lipidomic Markers of Health in Antibody-Positive Individuals

To test whether COVID-19 might lead to metabolic changes that persist even after asymptomatic infection or mild disease, we analyzed serum samples from 18 anti-S1-IgG+ and compared them with age- and sex-matched controls anti-S1-IgG-. Both groups were tested PCR-negative for asymptomatic SARS-CoV-2 infection on the same day of blood sampling. PCA showed no separation between the groups, and PLS-DA indicates a good separation. Based on ROC analysis, the resulted model indicates no relevant changes in the overall serum profile of metabolites and lipoproteins (Supplementary Figures S6A–C).

**FIGURE 6:**
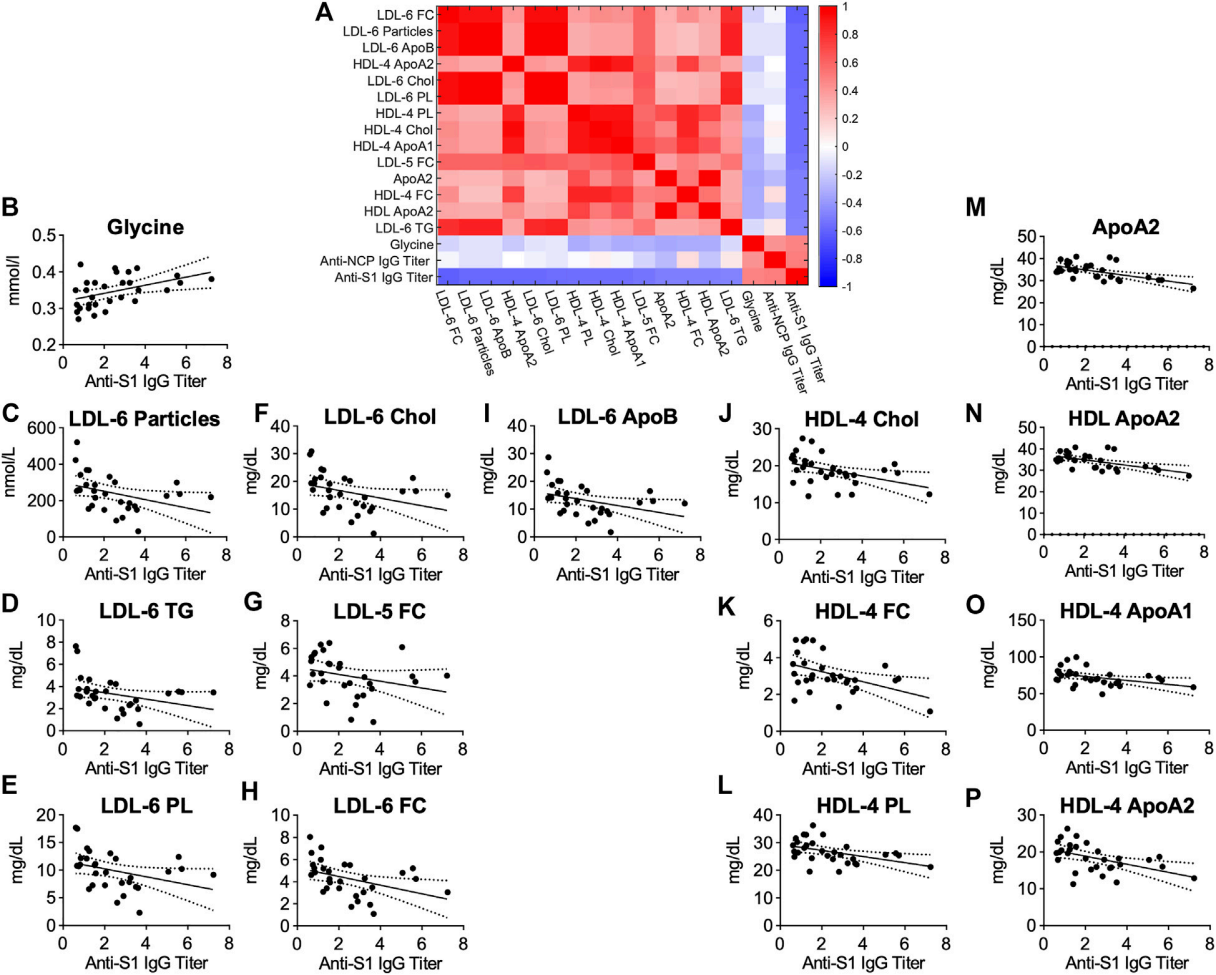
**(A**) Heatmap correlating metabolite and lipoprotein levels against anti-S1-IgG titer for individuals who tested positive for anti-SARS-CoV-2 antibodies (anti-S1-IgG+). **(B–P)** Spearman correlation plots for most significant individual metabolites or lipoproteins against anti-S1-IgG titer (*p*-values in Supplementary Table S2).

Possible links between metabolic serum profiles and antibody status were explored by examining associations between antibody titers in anti-S1-IgG+ samples. Antibody-titers against SARS-CoV-2 correlated inversely with small-sized LDL-6 particles and LDL-6 bound triglycerides, cholesterol, esterified cholesterol, and phospholipids. Similarly, several lipids bound in small-sized HDL-4 were negatively correlated (Figure 6). A strong positive correlation of antibody-titers was found for the amino acid glycine, a marker of metabolic health (Figure 6B, *p* = 0.0062, *r* = 0.4878, Supplementary Table S2). This signature differs from that observed for COVID-19 patients and may suggest an association of metabolic health and aznti-SARS-CoV-2 IgG antibody-titers in individuals after asymptomatic infection/mild disease.

The different signatures observed for COVID-19 patients and SARS-CoV-2 antibody-positive individuals are also seen in a direct comparison of the groups. For this, we focussed on key metabolites and lipoproteins that were significantly altered in any one of the cohorts. Supplementary Figure S7 shows that pyruvic acid and glucose were elevated in both ICU cohorts in comparison with SARS-CoV-2 antibody-positive and -negative individuals. Especially apolipoproteins, HDL, as well as some of their subfractions were decreased in the ICU cohorts. In contrast to that, VLDL-5 TG was increased.

## Discussion

The emergence of COVID-19 has rapidly developed into a global health crisis affecting health systems to an unprecedented extent. Primarily, a respiratory virus, SARS-CoV-2 affects multiple organ systems throughout the body, including the cardiovascular system, the kidney, and the liver. Although these organs are tightly involved and affected by metabolic processes, studies that target metabolism in COVID-19 have been scarce and looked at differences between COVID-19 patients during infection and HCs (Bruzzone et al., 2020; Kimhofer et al., 2020; Shen et al., 2020; Wu et al., 2020). Here, we have investigated metabolic changes during and after COVID-19 using ^1^H NMR spectroscopy of serum samples and compared them with not only HCs but also a cohort of critically ill CS patients.

The metabolic profile that we observe for COVID-19 compared with controls is largely in agreement with those previously published for cohorts in Spain and Australia (Bruzzone et al., 2020; Kimhofer et al., 2020). In general, several markers dysregulated in COVID-19 were reported earlier by others and in a preliminary study of ourselves (Schmelter et al., 2021), including increased levels of phenylalanine, glucose, and formic acid, as well as reduced levels of glutamine, histidine, and lactic acid. In opposite to the Spanish cohort, we do not see changes in ketone bodies, but the patients in our COVID-19 cohort did not receive glucose infusions, which is a likely cause for this difference, also reflected in lower glucose levels in our results. Reduced levels of glutamine, histidine, and lactic acid are indicative of metabolic suppression in COVID-19 patients supporting results of previous studies (Bruzzone et al., 2020; Kimhofer et al., 2020; Thomas et al., 2020).

The comparison of a critically ill COVID-19 intensive care cohort with other intensive care patients is important to answer the question of whether the previously described metabolic signature of COVID-19 is specific for this disease. HCs are subject to different environmental influences compared with ICU patients, eat a normal diet and engage in physical activities. For two cohorts with different diseases, but both treated in the same intensive care unit, the external factors are clearly more comparable. Changes in the metabolic profile are accordingly of greater relevance for the respective disease. The comparison of COVID-19 with critically ill ICU CS patients showed still a deeply disturbing lipoprotein profile typical for dyslipidemia that was more pronounced in COVID-19. Interestingly, lower levels of cholesterol and apolipoprotein A2 as well as in the HDL-bound form were found in CS patients compared with COVID-19. In contrast, samples from SARS-CoV-2 antibodies-positive but non-hospitalized individuals showed a remarkably different profile compared with COVID-19 patients. Interestingly, antibody-titers from those mild cases correlated with the amino acid glycine and lower levels of small-sized LDL and HDL subclasses.

Dyslipidemia was consistently found in previous studies using proteomics and NMR spectroscopy, indicating disturbed lipoprotein metabolism and increased cardiovascular risk in COVID-19 patients compared with HCs (Bruzzone et al., 2020; Kimhofer et al., 2020; Shen et al., 2020; Wu et al., 2020). Our data show a severely disturbed lipoprotein profile with remarkably increased TG levels potentially contributing to atherosclerosis (Nordestgaard et al., 2007; Nordestgaard and Varbo, 2014). Strikingly, this severely dyslipidemic profile is conserved in large parts in comparison with CS patients. Moreover, subfraction analysis shows that COVID-19 increases predominantly small-sized VLDL subclasses (VLDL-2, -3, and -4), which are more atherogenic than larger particles (Hodis et al., 1994; Carmena et al., 2004). Ample evidence suggests IDL and small VLDL remnants that are increased in COVID-19 as an important, independent risk factor for cardiovascular diseases and major cardiac injuries (Nordestgaard and Tybjaerg-Hansen, 1992; Carmena et al., 2004; Joshi et al., 2016; Pastori et al., 2018; Saeed et al., 2018; Borén et al., 2020).

Time course analysis confirmed an altered lipoprotein profile for COVID-19. Although COVID-19 and CS samples were different over a 7-day time course, there is considerable inter-patient variation in the development of time courses. Nevertheless, there are several markers that were different between COVID-19 and CS, also in longitudinally collected samples over 1 week. Specifically, this includes smaller-sized VLDL fractions (VLDL-2, 3, and -4) and IDL-bound lipids in cholesterol, triglycerides, and phospholipids, as well as ApoA2 and nearly all HDL ApoA2 fractions, which were different between COVID-19 and CS samples.

Importantly, cholesterol levels in VLDL, LDL, and HDL subclasses were affected in COVID-19. A recent systematic study shows that the formation of syncytia, a process that underlies viral entry *via* the ACE2 receptor, requires cholesterol (Sanders et al., 2021). From this point of view, we hypothesize that high cholesterol (and cholesterol ester) in certain VLDL and LDL subclasses (VLDL-4, -5, and LDL-1) favors membrane fusion events critical for viral infection. This would also explain recent successes with lipid apheresis as a treatment for long-COVID syndrome patients (Ärzteblatt DÄG Redaktion Deutsches, 2020; Bornstein et al., 2021).

Our analysis and those published earlier do not yet provide an answer to the question of whether the observed signature is a consequence of the disease or a profile that favors the development of severe COVID-19. Time courses were also not conclusive in this respect, although severe cases seem to correlate with high lipoprotein levels, often linked to high BMI. Overall, we suggest that high triglycerides and cholesterol in LDL and VLDL favor the development of a severe course of the COVID-19 disease. We do not observe regeneration of the lipoprotein profile during treatment monitored for 1 week. Future studies will have to show whether a metabolic and lipoprotein signature correlates with complete recovery after a longer period.

Metabolic and lipoprotein profiles from asymptomatic anti-SARS-CoV-2 antibody-positive patients without acute infection were similar to those of antibody-negative HCs. Those samples did not show the signature observed for ICU-treated COVID-19 patients. This points toward a greater level of metabolic health in individuals who were only mildly affected by COVID-19 symptoms. It is conceivable that a certain lipoprotein profile is a prerequisite for developing severe COVID-19, although this is not fully proven by these data. It is nevertheless likely that the development of COVID-19 is favored by a lipoprotein profile high in LDL and VLDL triglycerides and VLDL cholesterol.

Interestingly, we found significant associations of metabolic signatures with anti-S1-IgG antibody-titers. Interestingly, we observed correlations of anti-S1-IgG antibody-titers with glycine and lipoprotein fractions. Glycine is known to be reduced in patients with typical metabolic disorders (e.g., obesity, diabetes mellitus type II, and nonalcoholic fatty liver disease) (Alves et al., 2019), has been shown to reduce cholesterol and lipid levels in rodents (Park et al., 1999), and correlates inversely with risk of acute myocardial infarction in patients with stable angina pectoris (Ding et al., 2016). Therefore, glycine can potentially be considered as a marker of metabolic health (Alves et al., 2019) and is currently tested as a therapeutic agent for metabolic syndrome, atherosclerosis, and even COVID-19 (ClinicalTrials.gov identifier: NCT04443673). Older studies have shown that glycine is linked to the proliferation of lymphocytes and possibly increases antibody production (Duval et al., 1991; Li et al., 2007). The inverse correlation of antibody-titers with LDL, especially of smaller sizes (LDL-3–6), can be seen as an indicator of increased metabolic health in individuals with high titers against SARS-CoV-2. However, at this point, we cannot determine cause and effect from the available data. Possibly, individuals in a healthy metabolic state are more likely to mount an effective immune response to SARS-CoV-2, resulting in higher antibody-titers after mild or asymptomatic infection or alternatively, a certain level of “bad” lipoproteins favors viral infection of cells. These findings deserve further investigation as potential markers for individuals who are more likely to mount an adequate immune reaction to SARS-CoV-2 compared with those who are at risk of developing more severe disease symptoms. The knowledge of a specific lipid profile that is particularly susceptible to a severe course of COVID-19 would provide the opportunity to protect appropriate individuals at an early stage and to highlight them as a risk group.

The limitations of this study arise mainly from the small size of the COVID-19 and CS cohorts. However, our signature is largely in agreement with those published previously. Differences between COVID-19 and CS ICU patients must not be overinterpreted, considering the small sample size. One may also question the choice of CS ICU patients to compare with COVID-19. This is well justified because CS is also often associated with respiratory problems (Masip et al., 2018). A comparison with other acute respiratory distress syndrome patients would be preferable, but those patients are rare, and we did not have access to such a control group. However, our study includes a longitudinal sample collection that provides additional information on disease progression and is, to the best of our knowledge, so far, unique.

Another limitation is that the anthropometric data from the HCs we had access to were not optimally matched to the ICU cohorts. For example, compared with the COVID-19 patients, the HCs are significantly younger and have a higher BMI. However, the choice of HCs becomes significantly more difficult in a higher age group, and the difference in age was just about one decade. The HC group will also reflect environmental influences, such as diet and physical activity. This further justifies the need to also compare against another group of ICU patients where differences in metabolic profiles can be attributed to the different types of disease.

In conclusion, we show that the metabolic profile of COVID-19 patients is critically disrupted, showing typical signs of a pre-cachectic catabolic state, hepatic damage, and severe dyslipidemia even compared with ICU patients with CS. We show for a small cohort that this differentiation holds over a time course of 1 week. Curiously, antibody-titers after mild infection with SARS-CoV-2 correlate with markers of metabolic health. Taken together, these results underline the severity of changes in amino acid and lipoprotein metabolism and further support the relevance of metabolic disruption by COVID-19. At this point, we assume that the signature that we and others have identified is suitable to predict the severity of a COVID-19 infection, probably because it favors mechanisms of viral infection.

## Data Availability

The raw data supporting the conclusion of this article will be made available by the authors without undue reservation.
