# Supplementary material for: Metabolic and Lipidomic Markers Differentiate COVID-19 From Non-Hospitalized and Other Intensive Care Patients
Source: Front Mol Biosci. 2021 Dec 2;8:737039. doi: 10.3389/fmolb.2021.737039 (PMC8686182; doi:10.3389/fmolb.2021.737039)
Supplement: Supplementary file 1 [file DataSheet1.pdf]

Supplementary Material

Metabolic and lipidomic markers differentiate COVID-19 from non-hospitalized and other intensive care patients

Franziska Schmelter[1,6†], Bandik Föh[1,2†], Alvaro Mallagaray[3], Johann Rahmöller[1,9], Marc Ehlers[1], Selina Lehrian[1], Vera von Kopylow[1], Inga Künsting[1], Anne Sophie Lixenfeld[1], Emily Martin[1], Mohab Ragab[1], Roza Meyer-Saraei[7,8], Fabian Kreutzmann[7,8], Ingo Eitel[7,8], Stefan Taube[4], Nadja Käding[5], Eckard Jantzen[6], Tobias Graf[7,8††], Christian Sina[1,2††], Ulrich L Günther[3††]

Table S1. Descriptive statistics for metabolites and lipoproteins in COVID-19, HC, and CS cohorts**.** For each cohort, mean and standard deviation are shown. P-Values show unadjusted p-values from unpaired t-tests.

							**COVID-19 *vs.* HC**		**COVID-19 *vs.* CS**		**CS *vs.* HC**	
	**COVID-19**		**HC**		**CS**		**Change in %**	**P value**	**Changes in %**	**P value**	**Change in %**	**P value**
	mean	SD	mean	SD	mean	SD						
**Metabolites**												
Alanine (mmol/l)	0.43	0.14	0.53	0.10	0.38	0.23	-19.6	*0.001207*	13.9	0.59863	-29.4	*0.000005*
Creatine (mmol/l)	0.08	0.08	0.03	0.04	0.05	0.07	184.3	*0.000672*	68.4	0.134926	68.8	0.126552
Creatinine (mmol/l)	0.12	0.04	0.09	0.02	0.11	0.06	39.2	*0.000003*	3.5	0.798795	34.4	*<0,000001*
Glutamine (mmol/l)	0.55	0.12	0.71	0.11	0.61	0.24	-22.9	*0.000006*	-9.6	0.299422	-14.7	*0.000557*
Glycine (mmol/l)	0.40	0.15	0.35	0.08	0.29	0.12	12.1	0.112016	35.8	0.026379	-17.4	*0.008796*
Histidine (mmol/l)	0.06	0.01	0.10	0.02	0.07	0.03	-37.2	*<0,000001*	-9.7	0.278744	-30.4	*<0,000001*
Isoleucine (mmol/l)	0.09	0.03	0.07	0.02	0.09	0.05	24.8	0.017030	8.4	0.587356	15.1	0.038670
Leucine (mmol/l)	0.12	0.05	0.14	0.04	0.12	0.06	-13.8	0.147592	3.3	0.842568	-16.5	0.032872
Phenylalanine (mmol/l)	0.11	0.04	0.08	0.02	0.08	0.04	30.6	*0.000148*	33.7	0.029756	-2.3	0.779637
Tyrosine (mmol/l)	0.06	0.02	0.07	0.02	0.07	0.03	-16.1	0.061054	-20.8	0.118895	6.0	0.445455
Valine (mmol/l)	0.28	0.07	0.29	0.06	0.25	0.10	-5.8	0.307764	10.4	0.393469	-14.7	*0.005767*
Acetic acid (mmol/l)	0.02	0.04	0.01	0.02	0.06	0.28	98.5	0.107327	-65.8	0.274587	480.4	*0.000139*
Formic acid (mmol/l)	0.03	0.02	0.01	0.01	0.02	0.02	115.2	*0.004080*	25.4	0.501467	71.6	0.014801

Lactic acid (mmol/l)	1.99	0.72	4.43	1.48	1.79	1.59	-55.2	*<0,000001*	10.9	0.91487	-59.6	*<0,000001*
Glucose (mmol/l)	10.01	2.68	4.08	1.44	8.74	3.68	145.3	*<0,000001*	14.6	0.152078	114.1	*<0,000001*
Glutamine/Glutamate ratio	4.82	2.12	4.83	2.12	8.48	3.55	-0.2	0.987164	-43.2	*0.000405*	75.7	*<0,000001*
Lactic acid/Pyruvic Acid ratio	25.63	1.56	101.31	33.52	19.44	7.10	-74.7	*<0,000001*	31.8	0.247346	-80.8	*<0,000001*
Fischer's ratio	3.12	0.20	3.40	0.63	3.06	1.03	-8.1	0.134514	2.1	0.024105	-10.0	0.026230
**Lipoproteins**												
ApoB100 (mg/dl)	102.37	28.02	87.46	25.09	73.95	24.61	17.0	0.057028	38.4	*0.002642*	-15.4	0.021378
LDL/HDL	1.54	0.76	2.08	0.91	1.74	3.50	-26.1	0.049293	-11.7	0.702129	-16.3	0.096041
ApoB100/ApoA1	1.02	0.31	0.60	0.24	0.77	0.37	70.3	*<0,000001*	32.5	0.03618	28.5	*0.004005*
ApoB Particles (mmol/l)	1861.33	509.47	1590.24	456.15	1344.59	447.57	17.0	0.057051	38.4	*0.002644*	-15.4	0.021385
VLDL Particles (mmol/l)	309.35	79.95	159.96	92.08	187.18	70.85	93.4	*<0,000001*	65.3	*0.000168*	17.0	0.087113
IDL Particles (mmol/l)	195.04	77.35	103.49	49.75	102.00	64.37	88.5	*<0,000001*	91.2	*0.001085*	-1.4	0.716573
LDL Particles (mmol/l)	1399.94	564.82	1322.71	376.44	1042.19	396.53	5.8	0.527514	34.3	0.022561	-21.2	*0.001045*
LDL-1 Particles (mmol/l)	393.29	207.03	209.49	64.86	243.32	123.63	87.7	*<0,000001*	61.6	0.006626	16.1	0.021411
LDL-2 Particles (mmol/l)	192.71	62.84	195.37	63.51	218.54	74.20	-1.4	0.891056	-11.8	0.292636	11.9	0.207588
LDL-3 Particles (mmol/l)	184.23	117.09	182.45	74.02	143.23	74.83	1.0	0.941651	28.6	0.125851	-21.5	0.011993

LDL-4 Particles (mmol/l)	135.55	150.40	178.72	107.91	78.53	115.83	-24.2	0.212933	72.6	0.144626	-56.1	*0.000081*
LDL-5 Particles (mmol/l)	157.75	134.44	221.24	102.65	111.30	113.42	-28.7	0.052804	41.7	0.189765	-49.7	*0.000008*
LDL-6 Particles (mmol/l)	270.31	199.08	354.17	180.18	237.77	201.75	-23.7	0.134417	13.7	0.469488	-32.9	*0.003500*
Chol (mg/dl)	166.43	45.98	208.42	41.59	118.73	35.19	-20.1	*0.001428*	40.2	*0.00168*	-43.0	*<0,000001*
VLDL (mg/dl)	29.79	10.83	22.43	15.06	17.12	9.11	32.8	0.103585	74.0	*0.001224*	-23.7	0.232713
VLDL-1 Chol (mg/dl)	7.43	4.60	9.71	8.92	5.74	6.81	-23.5	0.386440	29.6	0.256175	-41.0	0.047072
VLDL-2 Chol (mg/dl)	4.43	1.52	3.41	2.72	2.29	1.79	30.0	0.205958	93.5	*0.000115*	-32.8	0.092050
VLDL-3 Chol (mg/dl)	5.67	3.02	3.68	2.78	2.21	2.15	54.2	0.021625	156.0	*0.000636*	-39.8	0.072188
VLDL-4 Chol (mg/dl)	8.90	2.98	4.19	2.50	4.14	3.30	112.5	*<0,000001*	115.3	*0.000715*	-1.3	0.407800
VLDL-5 Chol (mg/dl)	2.47	0.98	1.25	0.58	1.93	1.12	96.7	*<0,000001*	28.0	0.244815	53.7	*0.000002*
IDL (mg/dl)	20.73	8.45	12.98	7.94	9.53	9.24	59.7	*0.001954*	117.6	*0.001995*	-26.6	0.176692
LDL (mg/dl)	74.69	42.79	114.20	32.81	57.33	28.88	-34.6	*0.000226*	30.3	0.126116	-49.8	*<0,000001*
LDL-1 Chol (mg/dl)	29.78	18.46	22.03	7.12	17.59	10.20	35.2	*0.005222*	69.3	0.011104	-20.1	*0.015293*
LDL-2 Chol (mg/dl)	13.10	7.36	19.54	7.22	17.73	7.18	-33.0	*0.004295*	-26.1	0.07556	-9.3	0.173650
LDL-3 Chol (mg/dl)	10.20	9.37	17.08	7.68	8.64	6.04	-40.3	*0.004988*	18.1	0.402016	-49.4	*0.000002*
LDL-4 Chol (mg/dl)	6.68	10.13	15.58	9.51	4.08	8.15	-57.1	*0.002915*	63.7	0.285967	-73.8	*<0,000001*
LDL-5 Chol (mg/dl)	8.11	9.68	17.32	8.21	5.90	8.73	-53.2	*0.000474*	37.5	0.363769	-65.9	*<0,000001*
LDL-6 Chol (mg/dl)	16.37	13.16	22.80	10.95	13.07	12.24	-28.2	0.062560	25.3	0.307751	-42.7	*0.000114*
HDL (mg/dl)	44.96	11.51	59.47	15.65	38.43	15.66	-24.4	*0.002407*	17.0	0.126568	-35.4	*<0,000001*

HDL-1 Chol (mg/dl)	18.71	6.84	20.19	9.72	13.84	9.46	-7.3	0.609243	35.2	0.076123	-31.5	*0.004703*
HDL-2 Chol (mg/dl)	10.32	2.45	9.61	3.30	7.23	2.20	7.4	0.471804	42.6	*0.000238*	-24.7	*0.002135*
HDL-3 Chol (mg/dl)	8.28	2.36	10.40	2.70	6.75	2.16	-20.4	*0.010468*	22.5	0.034662	-35.1	*<0,000001*
HDL-4 Chol (mg/dl)	6.86	6.41	17.76	4.99	9.93	5.30	-61.3	*<0,000001*	-30.9	0.190377	-44.1	*<0,000001*
VLDL FC (mg/dl)	12.88	5.16	9.97	6.28	7.69	4.51	29.1	0.126588	67.4	0.003709	-22.9	0.206156
VLDL-1 FC (mg/dl)	2.35	1.92	4.02	3.72	1.21	2.13	-41.6	0.129537	94.0	0.044809	-69.9	*0.001178*
VLDL-2 FC (mg/dl)	2.59	0.99	1.57	1.35	1.39	0.92	65.3	0.012363	86.2	*0.000738*	-11.2	0.695323
VLDL-3 FC (mg/dl)	2.95	1.46	1.75	1.52	1.35	1.13	68.1	0.011125	117.7	*0.001326*	-22.8	0.412432
VLDL-4 FC (mg/dl)	4.63	1.78	1.90	1.26	2.14	1.75	144.1	*<0,000001*	116.4	*0.00123*	12.8	0.119650
VLDL-5 FC (mg/dl)	0.99	0.42	0.63	0.53	0.87	0.63	56.9	0.025738	13.7	0.623537	38.1	0.023975
IDL FC (mg/dl)	6.12	2.43	3.92	2.40	2.63	2.53	56.2	*0.003369*	132.4	*0.000719*	-32.8	0.076886
LDL FC (mg/dl)	27.24	12.93	32.41	8.76	22.18	8.90	-15.9	0.070340	22.8	0.113704	-31.6	*<0,000001*
LDL-1 FC (mg/dl)	8.70	4.68	6.36	2.03	5.88	3.06	36.9	*0.001978*	48.0	0.022739	-7.5	0.300410
LDL-2 FC (mg/dl)	4.74	2.19	5.79	2.25	5.94	2.46	-18.0	0.131886	-20.2	0.183143	2.7	0.858783
LDL-3 FC (mg/dl)	4.48	3.14	5.03	2.09	3.89	2.35	-10.9	0.417351	15.3	0.326756	-22.7	*0.005479*
LDL-4 FC (mg/dl)	2.83	2.96	4.48	2.24	1.82	3.03	-36.8	0.021793	55.8	0.199507	-59.4	*<0,000001*
LDL-5 FC (mg/dl)	2.64	2.67	4.46	1.91	2.25	3.03	-40.9	*0.003359*	17.4	0.531936	-49.7	*0.000002*
LDL-6 FC (mg/dl)	3.48	2.87	5.63	2.44	3.45	3.26	-38.2	*0.005544*	1.0	0.790193	-38.8	*0.000087*
HDL FC (mg/dl)	7.98	3.98	13.56	4.43	9.62	5.10	-41.1	*0.000064*	-17.0	0.44427	-29.0	*0.000055*
HDL-1 FC (mg/dl)	2.57	1.93	5.34	2.29	2.65	2.67	-51.8	*0.000110*	-3.1	0.903164	-50.3	*<0,000001*

HDL-2 FC (mg/dl)	1.98	0.71	2.14	0.91	1.47	0.86	-7.2	0.572573	34.6	0.031958	-31.1	*0.001324*
HDL-3 FC (mg/dl)	0.94	0.64	2.06	0.85	0.84	0.65	-54.5	*0.000021*	11.8	0.450484	-59.3	*<0,000001*
HDL-4 FC (mg/dl)	0.98	1.17	2.78	1.30	1.21	1.00	-64.6	*0.000012*	-19.0	0.580907	-56.4	*<0,000001*
TG (mg/dl)	187.92	46.97	157.16	105.95	124.18	58.05	19.6	0.323657	51.3	*0.000789*	-21.0	0.211051
VLDL TG (mg/dl)	114.95	48.34	100.25	76.49	63.52	48.18	14.7	0.517630	81.0	*0.001348*	-36.6	0.049472
VLDL-1 TG (mg/dl)	51.06	31.19	58.88	54.21	30.28	36.97	-13.3	0.625985	68.6	0.017696	-48.6	0.020399
VLDL-2 TG (mg/dl)	18.83	8.83	14.66	12.17	9.18	6.30	28.4	0.253410	105.1	*0.000546*	-37.4	0.073463
VLDL-3 TG (mg/dl)	18.80	8.94	11.75	8.68	8.68	6.26	60.0	*0.009125*	116.6	*0.000644*	-26.1	0.256869
VLDL-4 TG (mg/dl)	17.31	6.66	8.15	4.27	9.08	5.65	112.3	*<0,000001*	90.6	*0.001783*	11.4	0.077313
VLDL-5 TG (mg/dl)	4.88	1.11	2.57	0.83	3.94	1.29	89.9	*<0,000001*	23.8	0.057408	53.4	*<0,000001*
IDL TG (mg/dl)	17.01	6.61	17.95	17.82	7.91	6.15	-5.3	0.855813	115.1	*0.000152*	-56.0	0.018743
LDL TG (mg/dl)	47.47	19.38	19.75	7.63	32.49	15.02	140.4	*<0,000001*	46.1	0.011475	64.5	*<0,000001*
LDL-1 TG (mg/dl)	17.23	8.46	5.52	2.84	11.93	5.93	212.1	*<0,000001*	44.4	0.030652	116.1	*<0,000001*
LDL-2 TG (mg/dl)	6.73	3.50	2.59	0.87	4.23	2.22	160.3	*<0,000001*	59.2	0.009659	63.6	*<0,000001*
LDL-3 TG (mg/dl)	5.17	2.23	2.49	0.79	3.75	1.48	107.4	*<0,000001*	38.1	0.021338	50.2	*<0,000001*
LDL-4 TG (mg/dl)	6.97	4.42	2.27	1.46	4.03	2.84	207.2	*<0,000001*	72.8	0.019509	77.8	*0.000007*
LDL-5 TG (mg/dl)	5.28	2.62	2.65	1.47	3.34	2.01	99.0	*<0,000001*	58.1	0.014751	25.8	0.075399
LDL-6 TG (mg/dl)	6.78	1.83	4.43	2.27	4.77	2.55	52.9	*0.000797*	41.9	0.008616	7.7	0.590666
HDL TG (mg/dl)	19.15	7.74	12.67	5.15	11.64	7.04	51.2	*0.000174*	64.5	0.005374	-8.1	0.783238
HDL-1 TG (mg/dl)	8.06	3.98	4.57	2.49	4.45	3.52	76.5	*0.000042*	81.1	0.008929	-2.5	0.792152

HDL-2 TG (mg/dl)	4.04	1.61	2.29	1.05	2.46	1.71	76.4	*0.000001*	64.4	0.006826	7.3	0.249182
HDL-3 TG (mg/dl)	3.60	1.15	2.42	1.02	2.48	1.63	48.6	*0.000302*	45.5	0.018555	2.1	0.570755
HDL-4 TG (mg/dl)	3.40	0.78	3.58	1.26	2.66	1.13	-4.9	0.644202	28.2	0.030169	-25.8	0.006940
VLDL PL (mg/dl)	26.95	11.49	23.44	14.02	14.33	8.67	15.0	0.406695	88.0	*0.000883*	-38.9	*0.013540*
VLDL-1 PL (mg/dl)	6.97	4.46	9.28	8.28	4.21	3.37	-24.9	0.344723	65.6	0.016536	-54.7	*0.007273*
VLDL-2 PL (mg/dl)	4.43	2.30	3.87	2.89	2.07	1.49	14.3	0.521768	114.2	*0.000872*	-46.6	*0.012570*
VLDL-3 PL (mg/dl)	6.05	3.09	4.24	2.63	2.57	2.00	42.7	0.029216	135.7	*0.000778*	-39.5	0.029777
VLDL-4 PL (mg/dl)	8.67	2.75	4.40	2.07	4.60	2.78	97.3	*<0,000001*	88.7	*0.000881*	4.6	0.172132
VLDL-5 PL (mg/dl)	2.49	1.28	1.62	0.65	2.03	1.23	53.3	*0.000213*	22.5	0.421991	25.1	*0.002240*
IDL PL (mg/dl)	8.19	2.64	9.15	5.41	2.59	2.47	-10.5	0.546953	216.2	*0.000002*	-71.7	*0.000002*
LDL PL (mg/dl)	54.94	24.69	64.46	16.01	41.48	16.86	-14.8	0.070372	32.4	0.04475	-35.6	*<0,000001*
LDL-1 PL (mg/dl)	19.73	11.19	12.62	3.66	12.40	6.39	56.3	*0.000007*	59.0	0.012279	-1.7	0.975649
LDL-2 PL (mg/dl)	9.22	3.38	10.85	3.54	10.72	3.89	-15.0	0.132558	-14.0	0.259271	-1.2	0.653038
LDL-3 PL (mg/dl)	7.38	5.46	9.62	3.84	6.26	3.39	-23.3	0.070911	17.8	0.326533	-34.9	*0.000088*
LDL-4 PL (mg/dl)	5.35	6.31	8.72	4.88	2.83	4.36	-38.7	0.030307	88.8	0.117985	-67.5	*<0,000001*
LDL-5 PL (mg/dl)	5.17	5.40	9.68	4.15	4.12	4.30	-46.6	*0.000810*	25.3	0.415115	-57.4	*<0,000001*
LDL-6 PL (mg/dl)	9.96	7.40	13.31	5.51	8.29	6.52	-25.2	0.057555	20.2	0.350257	-37.8	*0.000083*
HDL PL (mg/dl)	74.07	15.27	84.10	20.51	62.34	21.24	-11.9	0.104254	18.8	0.055888	-25.9	*0.000014*
HDL-1 PL (mg/dl)	26.47	8.60	25.32	12.42	19.59	10.68	4.6	0.755473	35.1	0.042246	-22.6	0.047582
HDL-2 PL (mg/dl)	18.69	3.72	15.24	5.07	13.42	3.57	22.7	0.024289	39.3	*0.000117*	-11.9	0.132553

HDL-3 PL (mg/dl)	15.94	3.61	16.95	4.25	13.17	4.00	-6.0	0.430053	21.0	0.022157	-22.3	*0.000133*
HDL-4 PL (mg/dl)	12.97	8.24	25.97	5.05	16.88	7.25	-50.0	*<0,000001*	-23.2	0.199888	-35.0	*<0,000001*
ApoA1 (mg/dl)	104.15	20.70	152.53	25.99	102.27	25.68	-31.7	*<0,000001*	1.8	0.629963	-33.0	*<0,000001*
HDL ApoA1 (mg/dl)	104.17	23.55	153.01	28.47	103.84	25.33	-31.9	*<0,000001*	0.3	0.799226	-32.1	*<0,000001*
HDL-1 ApoA1 (mg/dl)	28.86	11.51	33.12	17.80	25.80	17.22	-12.9	0.421547	11.9	0.457709	-22.1	0.068653
HDL-2 ApoA1 (mg/dl)	19.35	4.58	21.36	5.69	16.11	5.72	-9.4	0.242913	20.2	0.062705	-24.6	*0.000146*
HDL-3 ApoA1 (mg/dl)	24.41	5.01	27.98	6.51	21.21	4.57	-12.7	0.069745	15.1	0.030979	-24.2	*0.000007*
HDL-4 ApoA1 (mg/dl)	30.44	18.89	69.57	12.97	39.71	16.84	-56.3	*<0,000001*	-23.4	0.174768	-42.9	*<0,000001*
ApoA2 (mg/dl)	24.70	6.75	33.68	5.50	18.96	7.22	-26.6	*<0,000001*	30.3	*0.014853*	-43.7	*<0,000001*
HDL ApoA2 (mg/dl)	27.92	6.43	33.86	5.27	21.25	7.40	-17.5	*0.000468*	31.4	*0.003966*	-37.2	*<0,000001*
HDL-1 ApoA2 (mg/dl)	3.86	1.09	3.57	1.83	2.39	1.47	8.2	0.585283	61.8	*0.003047*	-33.1	*0.009060*
HDL-2 ApoA2 (mg/dl)	4.48	0.71	4.30	1.33	2.62	1.07	4.3	0.636109	71.2	*0.000002*	-39.1	*<0,000001*
HDL-3 ApoA2 (mg/dl)	6.78	1.53	7.06	1.61	4.43	1.68	-4.1	0.560885	53.0	*0.00008*	-37.3	*<0,000001*
HDL-4 ApoA2 (mg/dl)	8.22	5.92	17.99	4.35	9.12	4.56	-54.3	*<0,000001*	-9.9	0.692616	-49.3	*<0,000001*
VLDL ApoB (mg/dl)	17.01	4.40	8.80	5.06	10.29	3.90	93.4	*<0,000001*	65.3	*0.000169*	17.0	0.087014
IDL ApoB (mg/dl)	10.73	4.25	5.69	2.74	5.61	3.54	88.4	*<0,000001*	91.2	*0.001082*	-1.4	0.717293
LDL ApoB (mg/dl)	76.99	31.06	72.75	20.70	57.32	21.81	5.8	0.527487	34.3	0.022559	-21.2	*0.001046*
LDL-1 ApoB (mg/dl)	21.63	11.39	11.52	3.57	13.38	6.80	87.7	*<0,000001*	61.6	0.006628	16.1	0.025375
LDL-2 ApoB (mg/dl)	10.60	3.46	10.75	3.49	12.02	4.08	-1.4	0.892490	-11.8	0.292876	11.9	0.207721
LDL-3 ApoB (mg/dl)	10.13	6.44	10.03	4.07	7.88	4.12	1.0	0.940301	28.6	0.125808	-21.5	0.011995

LDL-4 ApoB (mg/dl)	7.45	8.27	9.83	5.93	4.32	6.37	-24.2	0.213101	72.6	0.144721	-56.1	*0.000082*
LDL-5 ApoB (mg/dl)	8.68	7.39	12.17	5.65	6.12	6.24	-28.7	0.052775	41.7	0.190003	-49.7	*0.000008*
LDL-6 ApoB (mg/dl)	14.87	10.95	19.48	9.91	13.08	11.10	-23.7	0.134526	13.7	0.469536	-32.9	*0.003502*

Table S2. Spearman Correlation for metabolites and lipoproteins against the Anti-S1 IgG-titer in COVID-19, HC, and CS cohorts.

	Glycine	ApoA2	LDL-6 Particles	HDL-ApoA2	LDL-6 TG	LDL-6 Chol	LDL-5 FC	LDL-6 FC	LDL-6 PL	LDL-6 ApoB	HDL-4 Chol	HDL-4 FC	HDL-4 PL	ApoA1	ApoA2
Spearman r	0.5101	-0.4146	-0.5099	-0.4126	-0.3905	-0.5051	-0.4593	-0.5535	-0.4938	-0.5099	-0.4949	-0.4092	-0.4952	-0.5011	-0.5257
P value															
P (two-tailed)	0.0029	0.0183	0.0029	0.0189	0.0271	0.0032	0.0082	0.001	0.0041	0.0029	0.004	0.0201	0.004	0.0035	0.002

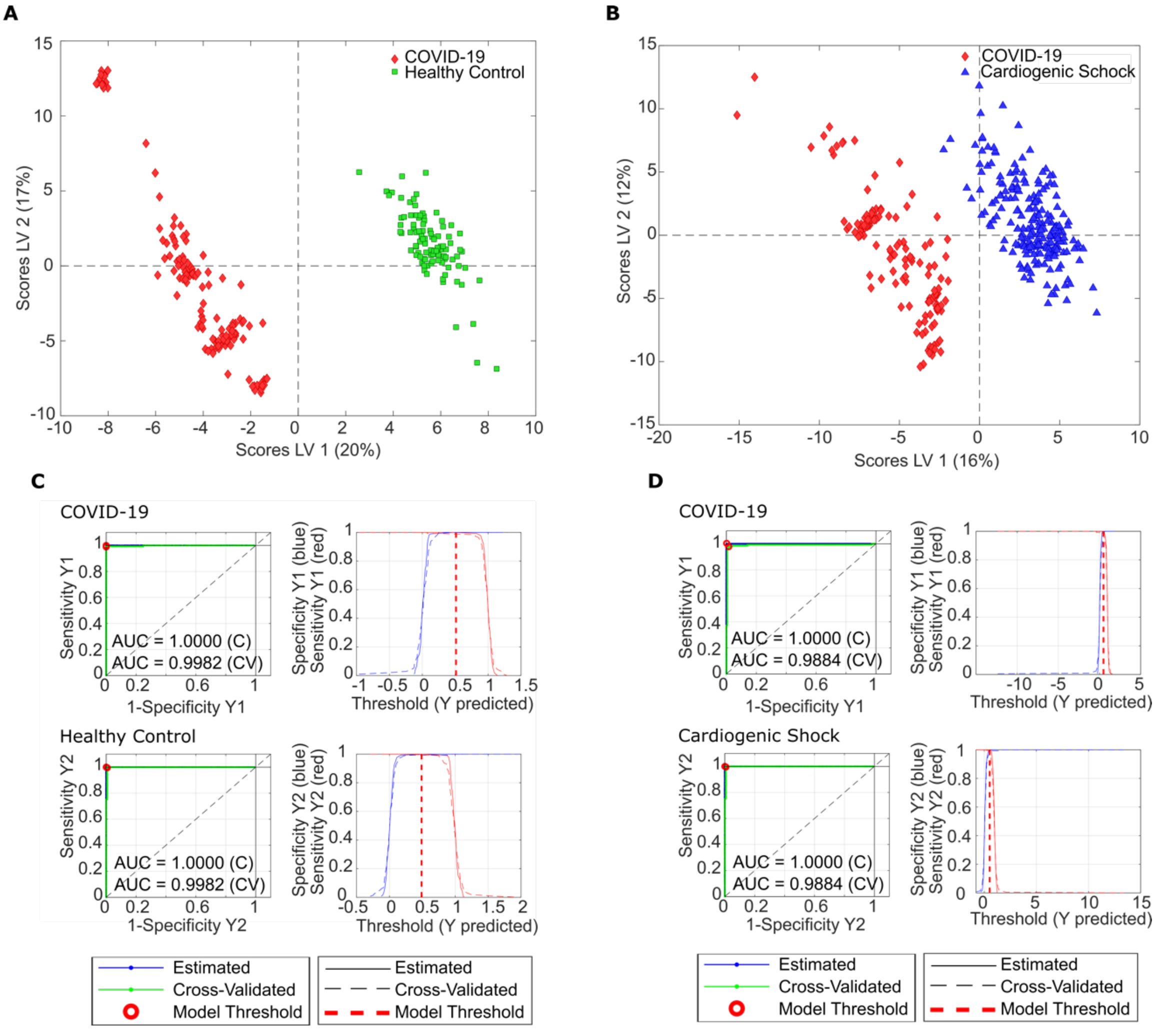

Supplementary Figure 1. **A** PLS-DA for COVID-19 *vs.* Healthy Controls. The ROC analysis in **C** indicates a significance for the separation of the two groups. **B** PLS-DA of COVID-19 *vs.* Cardiogenic Shock shows a good grouping for the two groups. A good separation model was validated by a ROC analysis (**D**).

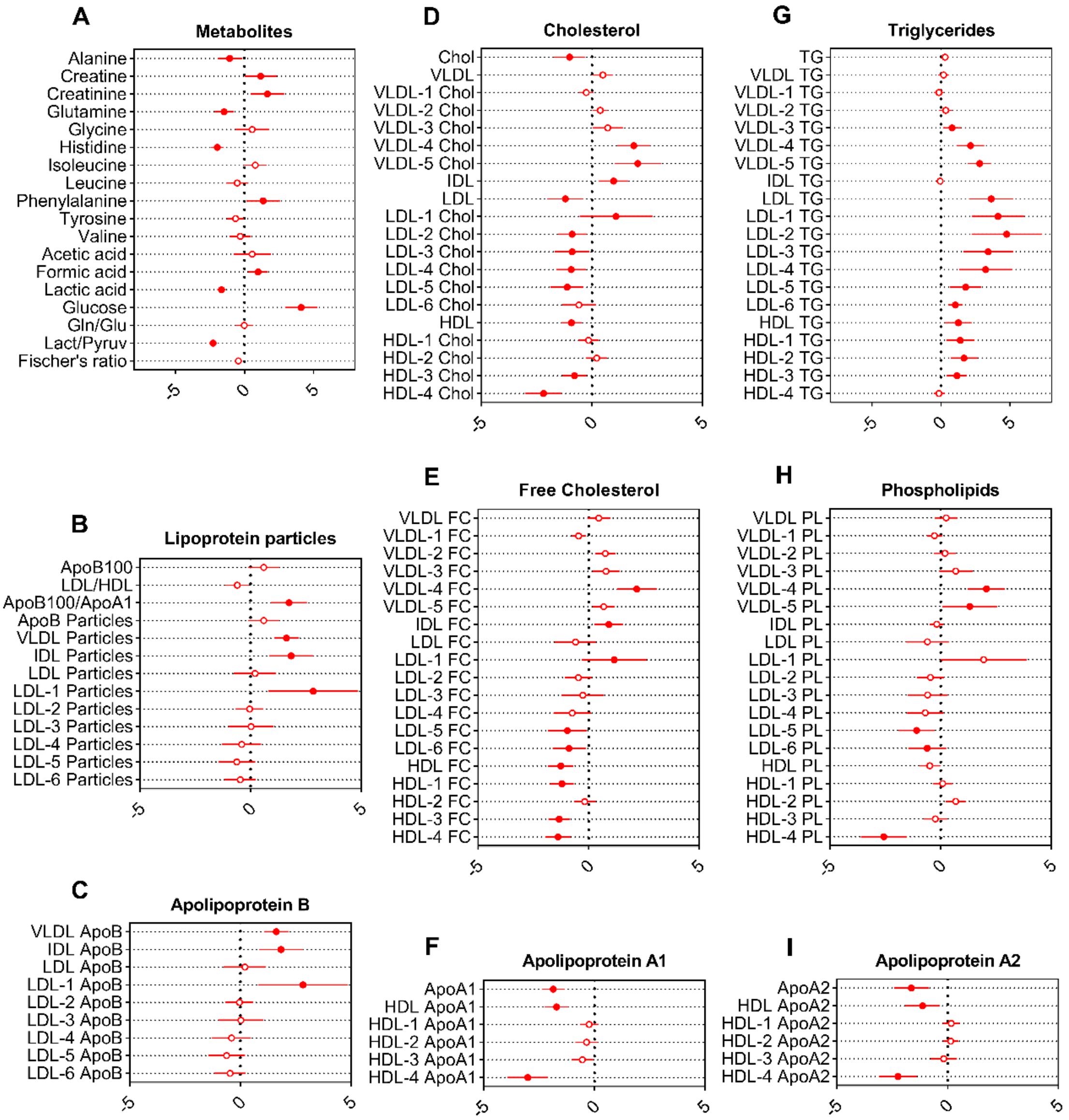

Supplementary Figure 2. Forest plots showing changes for groups of metabolites (**A**) and lipoprotein classes (**B-I**). The middle line indicates the reference average whereas circles on the horizontal axes show changes scaled by standard deviations. Red circles show changes for COVID-19 samples compared to healthy controls as the reference (vertical line). Statistically significant differences between COVID-19 to HC determined using the false discovery method of Benjamini, Krieger and Yekutieli (Q = 1%) are indicated by filled circles.

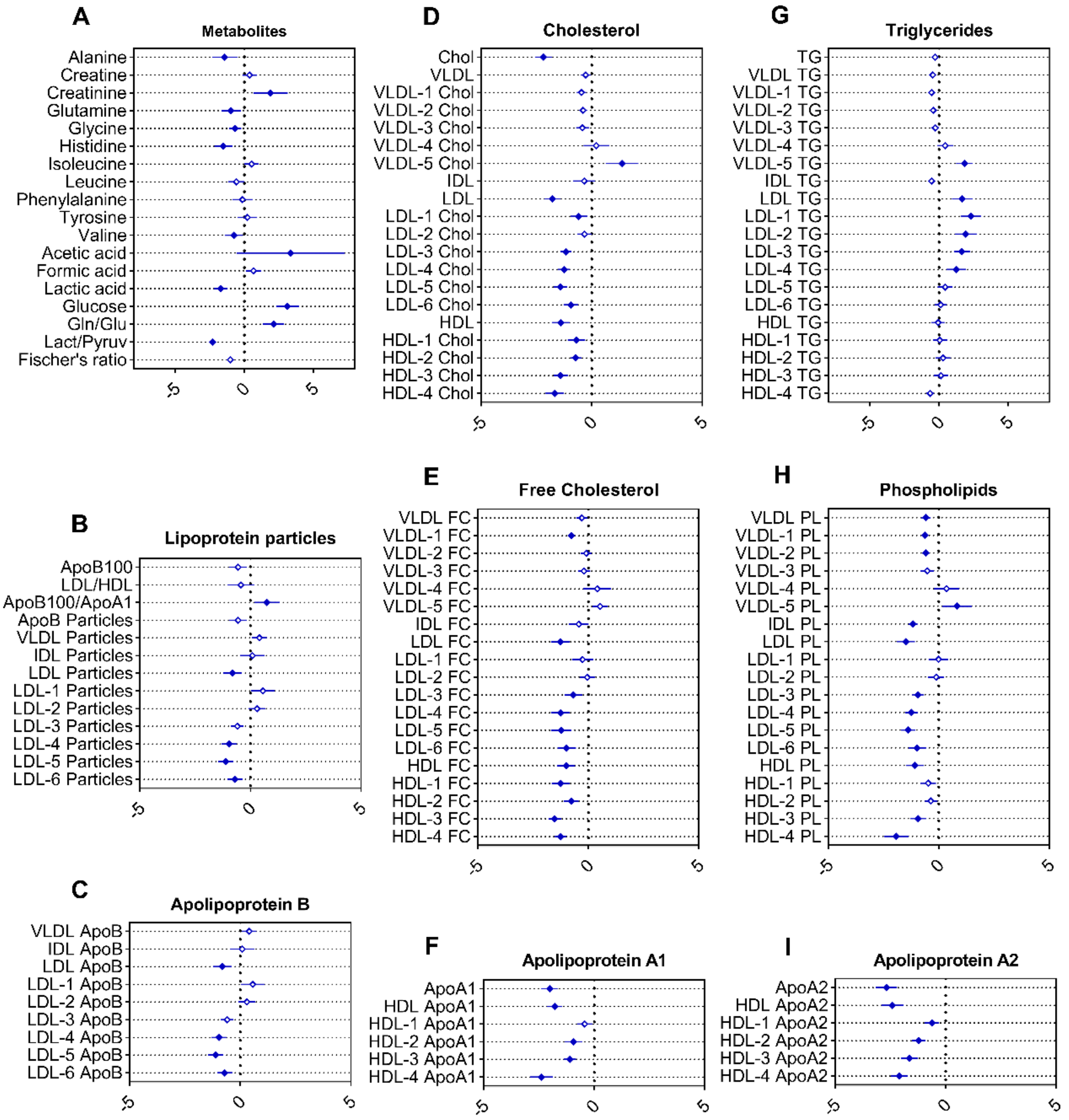

Supplementary Figure 3. Forest plots showing changes for groups of metabolites (**A**) and lipoprotein classes (**B-I**). The middle line indicates the reference average whereas diamonds on the horizontal axes show changes scaled by standard deviations. Blue diamonds show changes for CS samples compared to healthy controls as the reference (vertical line). Statistically significant differences between CS to HC determined using the false discovery method of Benjamini, Krieger and Yekutieli (Q = 1%) are indicated by filled diamonds.

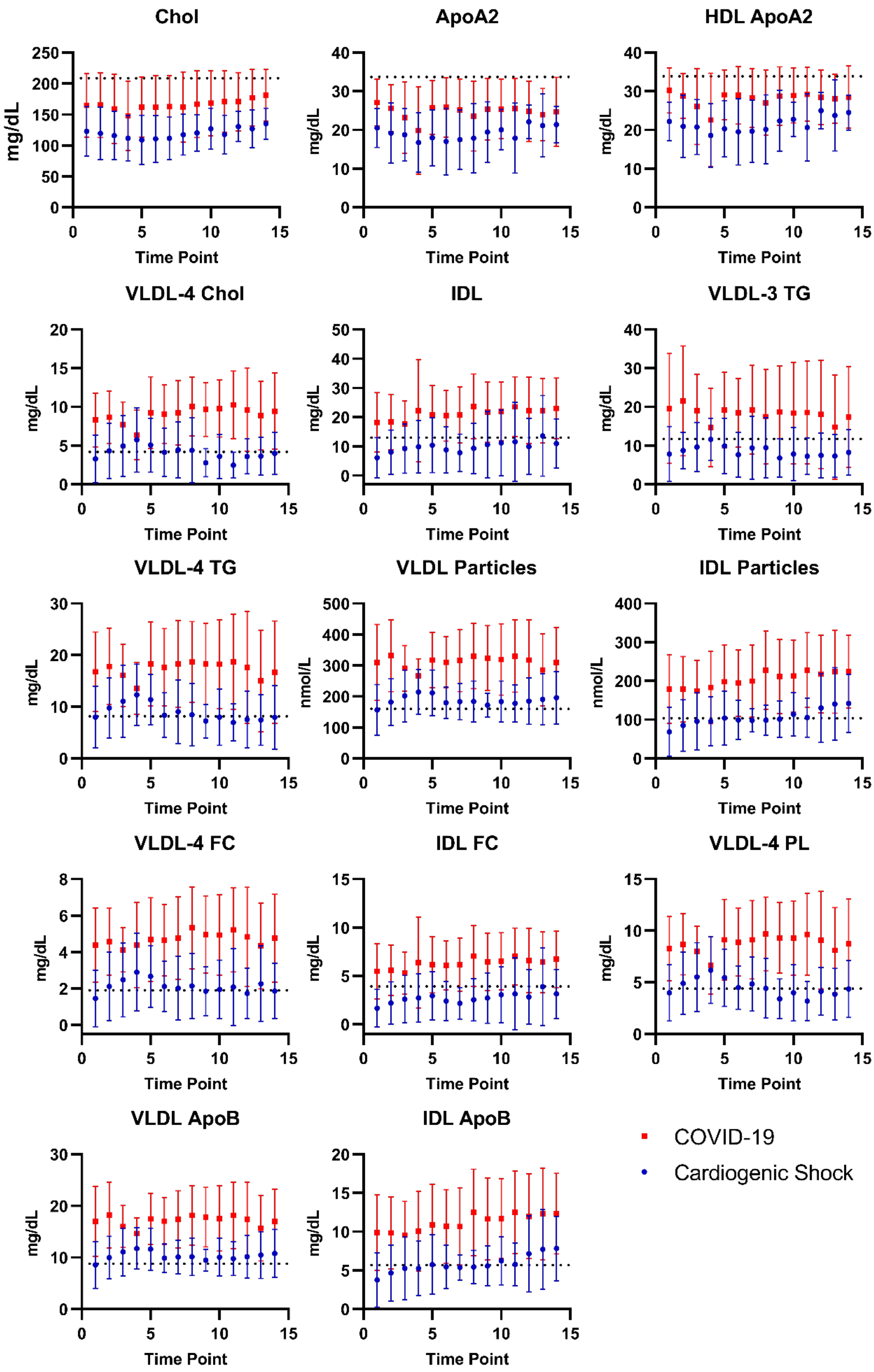

Chol
ApoA2
HDL ApoA2
VLDL-4 Chol
IDL
VLDL-3 TG
VLDL-4 TG
VLDL Particles
IDL Particles
VLDL-4 FC
IDL FC
VLDL-4 PL
VLDL ApoB
IDL ApoB
mg/dL
nmol/L
Time Point
COVID-19
Cardiogenic Shock

Supplementary Figure 4. Time courses of lipoproteins (mean and standard deviation) that are significant for the both comparison of COVID-19 to HC and CS allows to differentiate between COVID-19 (red) and cardiogenic shock (blue) showing a continuous progression for COVID-19; the dotted line represents the mean value for healthy controls.

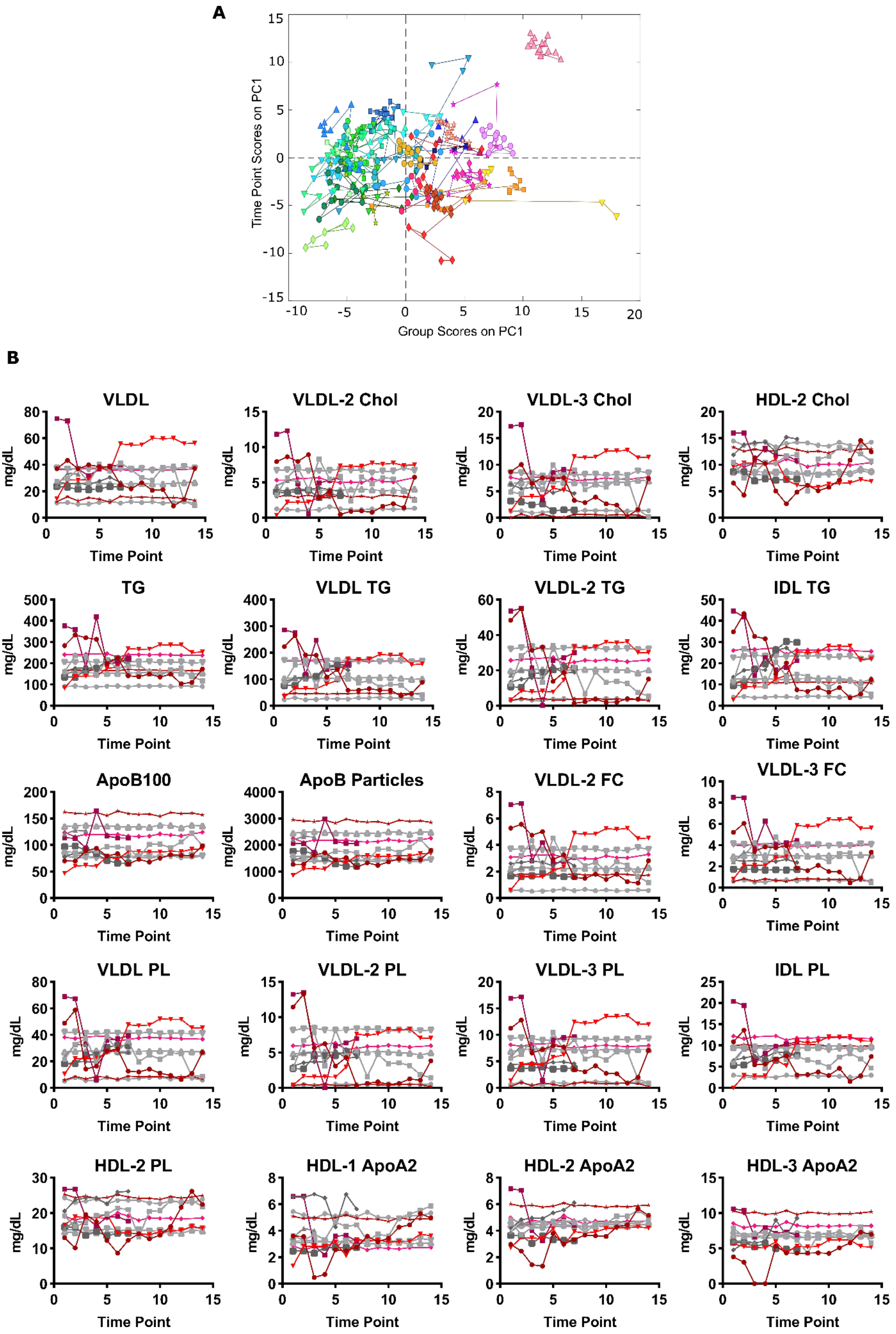

Supplementary Figure 5. **A** ASCA analysis of COVID-19 (warm colors in yellow and red) *vs.* cardiogenic shock (cold colors in blue and green) samples showing a separation between the groups in consideration of the

different groups and time points. **B** Time courses of lipoproteins for COVID-19 patients that are only significant for the comparison of COVID-19 *vs.* CS showing striking progressions over time for some patients (highlighted in red).

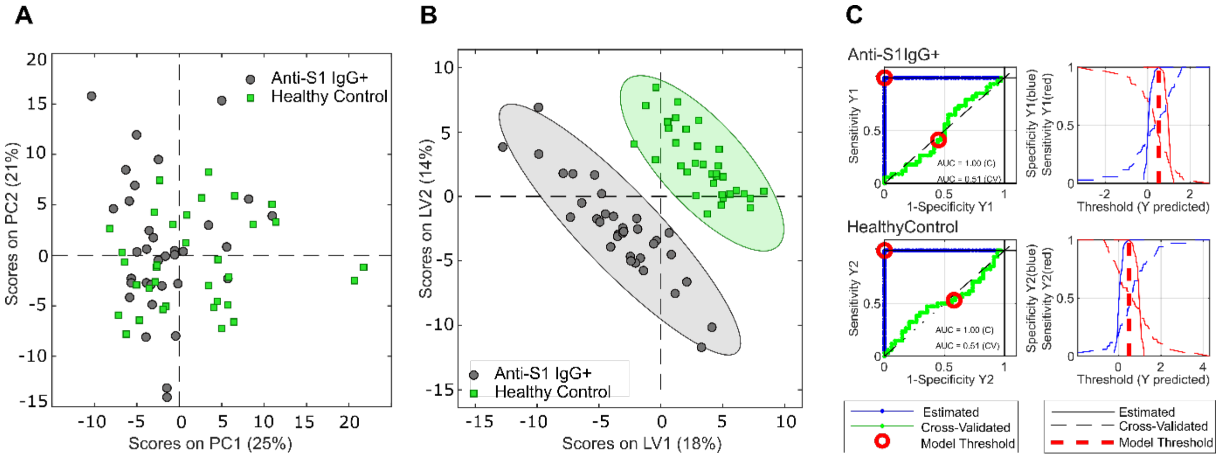

Supplementary Figure 6. **A** PCA and **B** PLS-DA for Anti-S1 IgG+ *vs.* age- and sex-matched Healthy Controls. The ROC analysis in **C** shows no significance for the separation of the two groups.

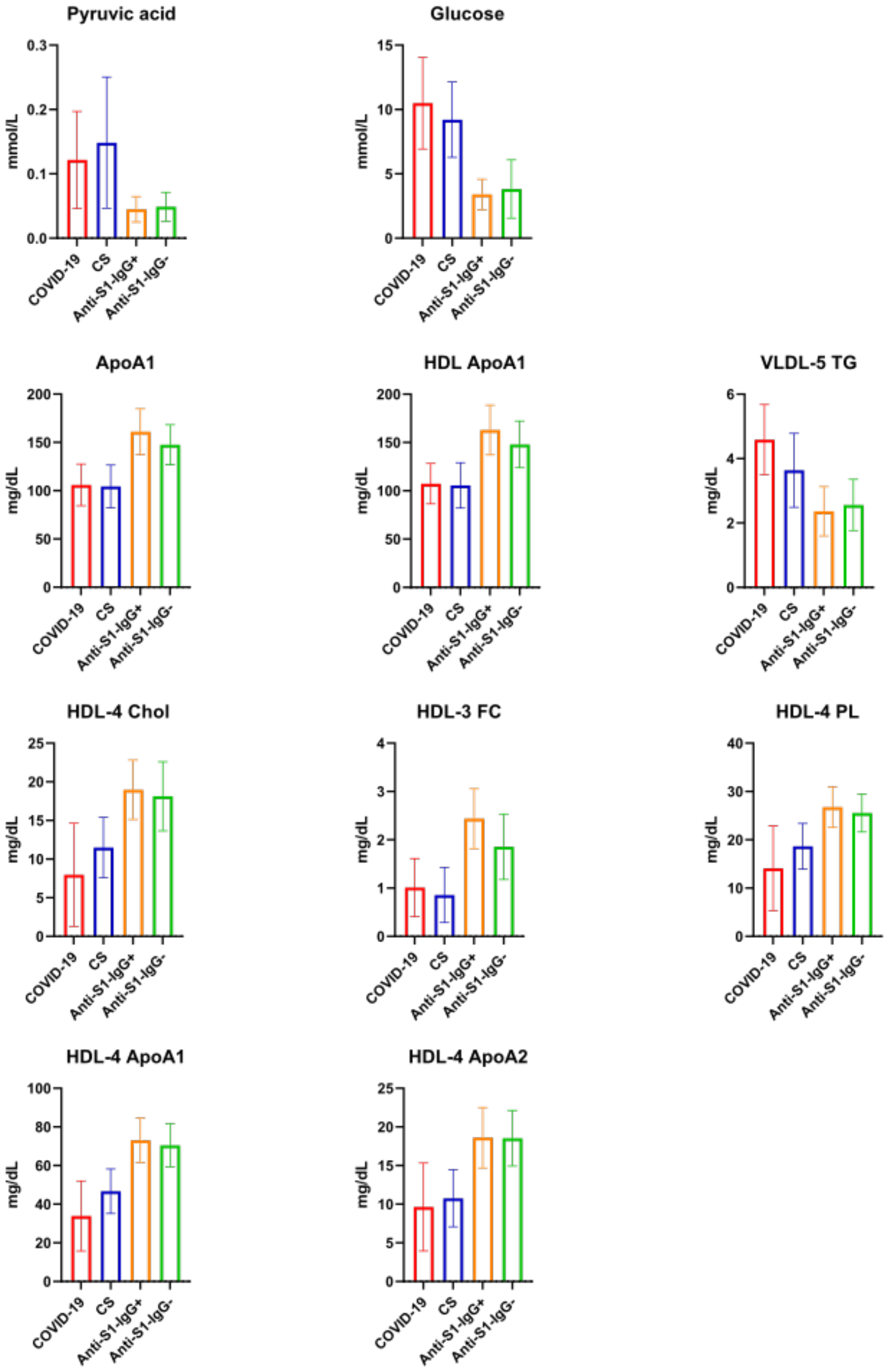

Supplementary Figure 7. Metabolites and lipoproteins significantly changed between (1) COVID-19 vs. Anti-SARS-CoV-2 IgG+ individuals (Anti-S1-IgG+), (2) COVID-19 vs. Anti-SARS-CoV-2 IgG-antibodies (Anti-S1-IgG-), (3) CS vs. Anti-S1-IgG+, (4) CS vs. Anti-S1-IgG determined using multiple unpaired t-test with the false discovery method of Benjamini, Krieger and Yekutieli (Q = 1%).
